# Society for Cardiovascular Magnetic Resonance 2019 Case of the Week series

**DOI:** 10.1186/s12968-020-00671-7

**Published:** 2021-04-01

**Authors:** Arun Dahiya, Charles Chao, John Younger, Julia Kar, Bryant M. Baldwin, Michael V. Cohen, Shane Joseph, Anam Chowdhry, Maria S. Figarola, Christopher Malozzi, M. Farhan Nasser, Yassar Nabeel, Rajiv Shah, J. Michael Kennen, Ashish Aneja, Sameh Khalil, Sara Ragab, Omnia Mohammed, Taher Moustafa, Ahmed Hamdy, Shimaa Ahmed, Ahmed Heny, Maha Taher, Madhusudan Ganigara, Arushi Dhar, Nilanjana Misra, Jafar Alzubi, Kurian Pannikottu, Ahmad Jabri, Vinayak Hedge, Anmar Kanaa’n, Joseph Lahorra, Dominique de Waard, David Horne, Santokh Dhillon, Aoife Sweeney, Christian Hamilton-Craig, V. S. Katikireddi, Allan J. Wesley, Chris Hammet, Jason N. Johnson, Sylvia S. M. Chen

**Affiliations:** 1 Royal Brisbane and Women’s Hospital, Brisbane, QLD Australia; 2 Griffith University School of Medicine, Gold Coast, QLD Australia; 3 Departments of Mechanical Engineering and Pharmacology, University of South Alabama, Mobile, AL USA; 4 Department of Cardiology, University of South Alabama, Mobile, AL USA; 5 Department of Radiology, University of South Alabama, Mobile, AL USA; 6 Department of Internal Medicine, Cleveland Clinic Akron General, Akron, OH USA; 7 Department of Internal Medicine, Division of Cardiology, Metrohealth Medical Center, Case Western Reserve University, Cleveland, OH USA; 8 Department of Radiology, Metrohealth Medical Center, Case Western Reserve University, Cleveland, OH USA; 9 Alfa Scan Radiology Center, Cardiovascular Imaging Department, Cairo, Egypt; 10 Division of Pediatric Cardiology, Cohen Children’s Medical Center of New York-Hofstra Northwell School of Medicine, Hempstead, NY USA; 11 Department of Cardiology, Cleveland Clinic Akron General, Akron, OH USA; 12 Department of Cardiothoracic Surgery, Cleveland Clinic Akron General, Akron, OH USA; 13 Faculty of Medicine, Dalhousie University, Halifax, NS Canada; 14 Isaac Walton Killam Children’s Hospital, Halifax, NS Canada; 15 Department of Rheumatology, The Prince Charles Hospital, Brisbane, QLD Australia; 16 Department of Cardiology, The Prince Charles Hospital, Brisbane, QLD Australia; 17 Department of Medical Imaging, The Prince Charles Hospital, Brisbane, QLD Australia; 18 Le Bonheur Children’s Hospital, Memphis, TN USA

**Keywords:** Cardiomyopathy, Cardiac tumor, MRI, Eosinophilic granulomatosis

## Abstract

The Society for Cardiovascular Magnetic Resonance (SCMR) is an international society focused on the research, education, and clinical application of cardiovascular magnetic resonance (CMR). The SCMR web site (https://www.scmr.org) hosts a case series designed to present case reports demonstrating the unique attributes of CMR in the diagnosis or management of cardiovascular disease. Each clinical presentation is followed by a brief discussion of the disease and unique role of CMR in disease diagnosis or management guidance. By nature, some of these are somewhat esoteric, but all are instructive. In this publication, we provide a digital archive of the 2019 Case of the Week series as a means of further enhancing the education of those interested in CMR and as a means of more readily identifying these cases using a PubMed or similar search engine.

**Supplementary information:**

**Supplementary information** accompanies this paper at 10.1186/s12968-020-00671-7.

## Introduction

Firstly, a huge thank you to our wonderful team of associate editors and reviewers for the Society for Cardiovascular Magnetic Resonance (SCMR) website “Case of the Week” series. This past year, the cases were predominantly from the United States, with international cases from Egypt, Canada and Australia. There was a mixture of adult and paediatric cases, showcasing the utility of cardiovascular magnetic resonance (CMR) in assessing and diagnosing disease. The importance of CMR in mass differentiation, particularly in the paediatric population where solitary masses/tumours are rare, was well demonstrated. Early CMR diagnosis was demonstrated despite seemingly negative testing by other methods including tissue biopsy. The use of a research tool in the clinical setting was also reported, giving a glimpse of its potential use in early identification of a pathological process and therefore help guide clinical management. Here are the SCMR Case of the Week series of 2019. Please continue to submit your instructive cases to: scmr.org/page/SubmitCase.

## Case 1: Early diagnosis of a systemic disease by CMR prevents complications

### Clinical history

A 19-year-old woman presented with a 2-week history of painful atraumatic left ankle swelling associated with painful plantar-flexion and petechial rash on the left foot. Six weeks prior to presentation, she experienced a flu-like illness with headache, fevers, myalgias, and athralgias. On examination, there was a non-blanching petechial rash over the dorsum of the left foot with associated edema and warmth, but no erythema. Peripheral pulses were normally palpable with normal capillary refill. Neurological exam of the foot was normal except for 4/5 power on plantar flexion due to pain. Ultrasound of the leg showed a thrombus in the lateral tarsal artery raising a possibility of an embolus.

**Fig. 1.**
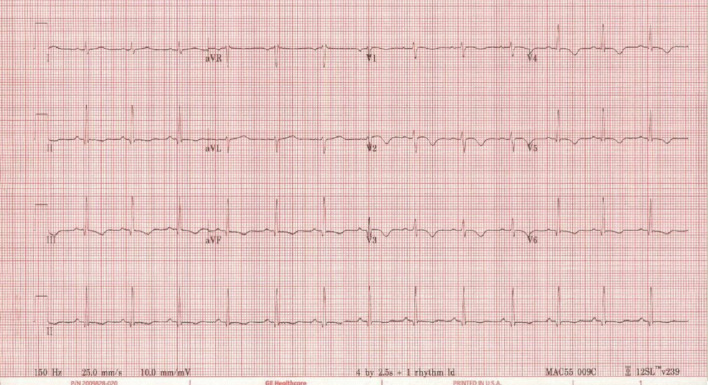
**Case 1.** 12 lead electrocardiogram (ECG). Sinus rhythm with T-wave inversion in most precordial leads (V2−V6) and some inferior limb leads (III and aVF)

Her past medical history was remarkable for mild childhood asthma that was associated with viral infections and exercise; and allergic rhinitis. She had no prior hospitalizations. Chest X-ray was unremarkable. Electrocardiogram (ECG) showed T-wave inversion in most precordial leads (V2–V6) and some inferior leads (III and aVF) (Fig. 1).

Routine blood work was unremarkable except for eosinophilia which was attributable to asthma. Inflammatory markers (erythrocyte sedimentation rate (ESR) 41 mm/h and C-reactive protein (CRP) 22 mg/l were mildly elevated. Troponin was negative. Transthoracic echocardiogram (TTE) performed to evaluate for a possible cardiac embolic source was unremarkable except for mildly increased left ventricular (LV) apical wall thickness (Additional file 1: Movie S1). Given the abnormal ECG and increased LV apical wall thickness, CMR was requested to assess for possible apical hypertrophic cardiomyopathy (HCM).

### CMR findings

3 T CMR (Siemens Healthineers, Erlangen Germany) showed normal biventricular size and systolic function with only minimal increase in the LV apical wall thickness (Additional file 2: Movie S2, Additional file 3: Movie S3, Additional file 4: Movie S4).

**Fig. 2:**
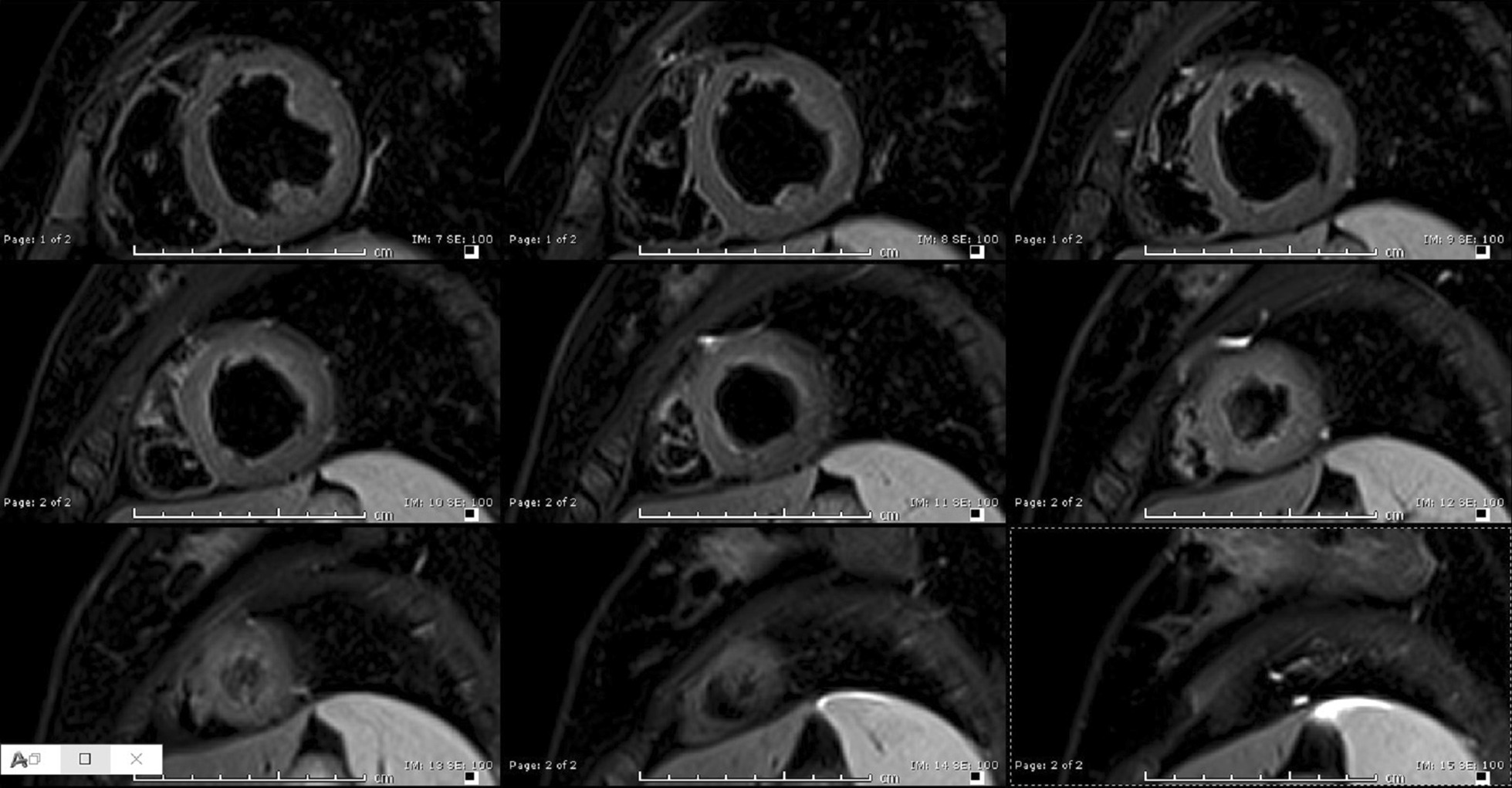
**Case 1.** T2-weighted short tau inversion recovery (STIR) imaging of the left ventricular (LV) short axis from base to apex. Diffuse increase in subendocardial signal intensity suggestive of subendocardial edema

The LV mass index was 64 g/m^2^, the LV end diastolic volume index was 73 ml/m^2^, with a low normal LV ejection fraction (LVEF) of 57%. The right ventricular (RV) size and systolic function were normal with an RV end diastolic volume index of 74 ml/m^2^ and RV ejection fraction (RVEF) of 51%. LV tissue characterization revealed subtle sub-endocardial abnormalities. T2-weighted (T2w) imaging showed somewhat diffuse sub endomyocardial edema in mid to distal LV segments (Fig. 2). Rest perfusion sequences in short axis and apical 4-chamber view showed mid to apical sub-endocardial hypo-perfusion (Additional file 5: Movie S5, Additional file 6: Movie S6).

**Fig. 3:**
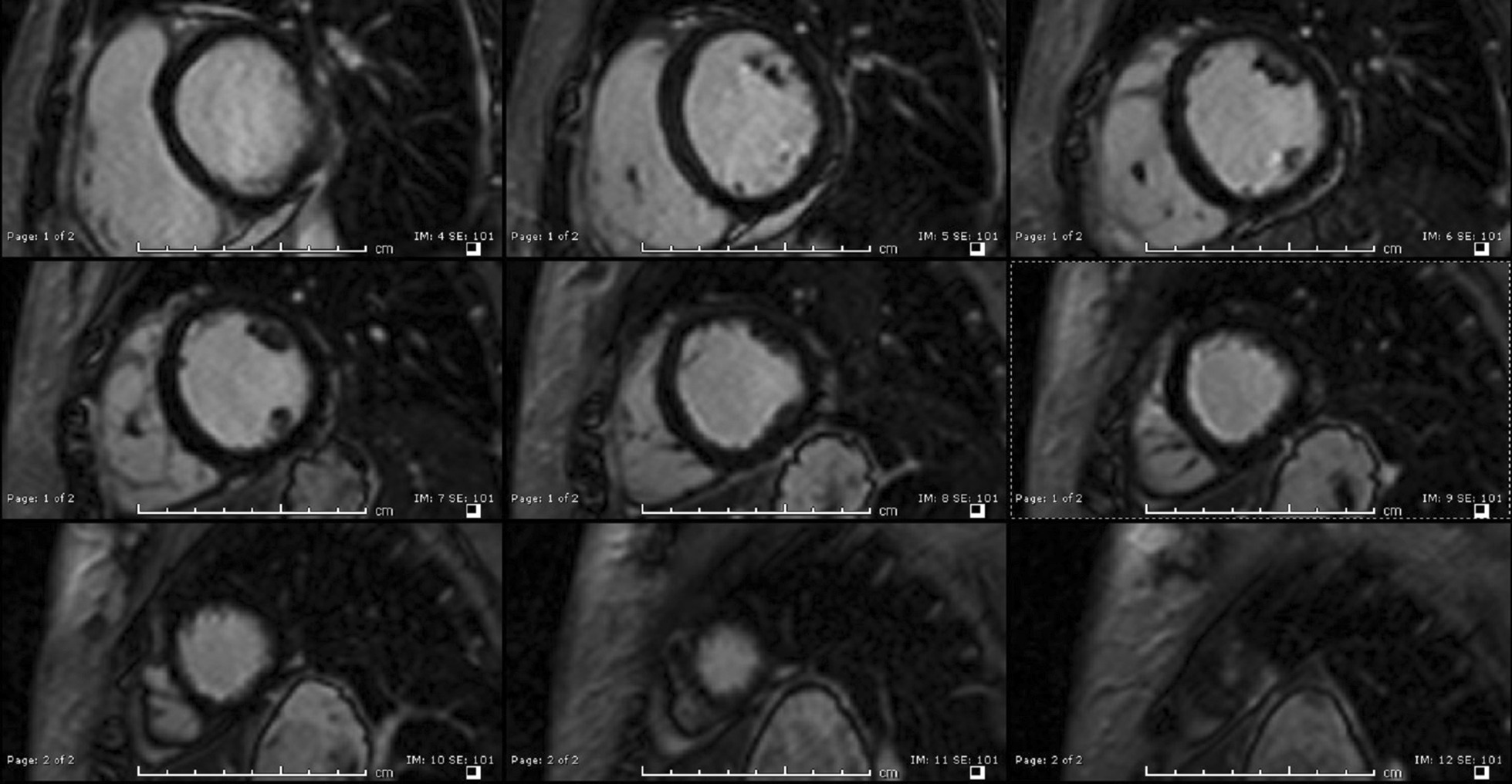
**Case 1.** Short axis stack T1 weighted (high resolution-magnitude) late gadolinium enhancement (LGE) imaging. Subendocardial enhancement in the mid to distal LV cavity

**Fig. 4:**
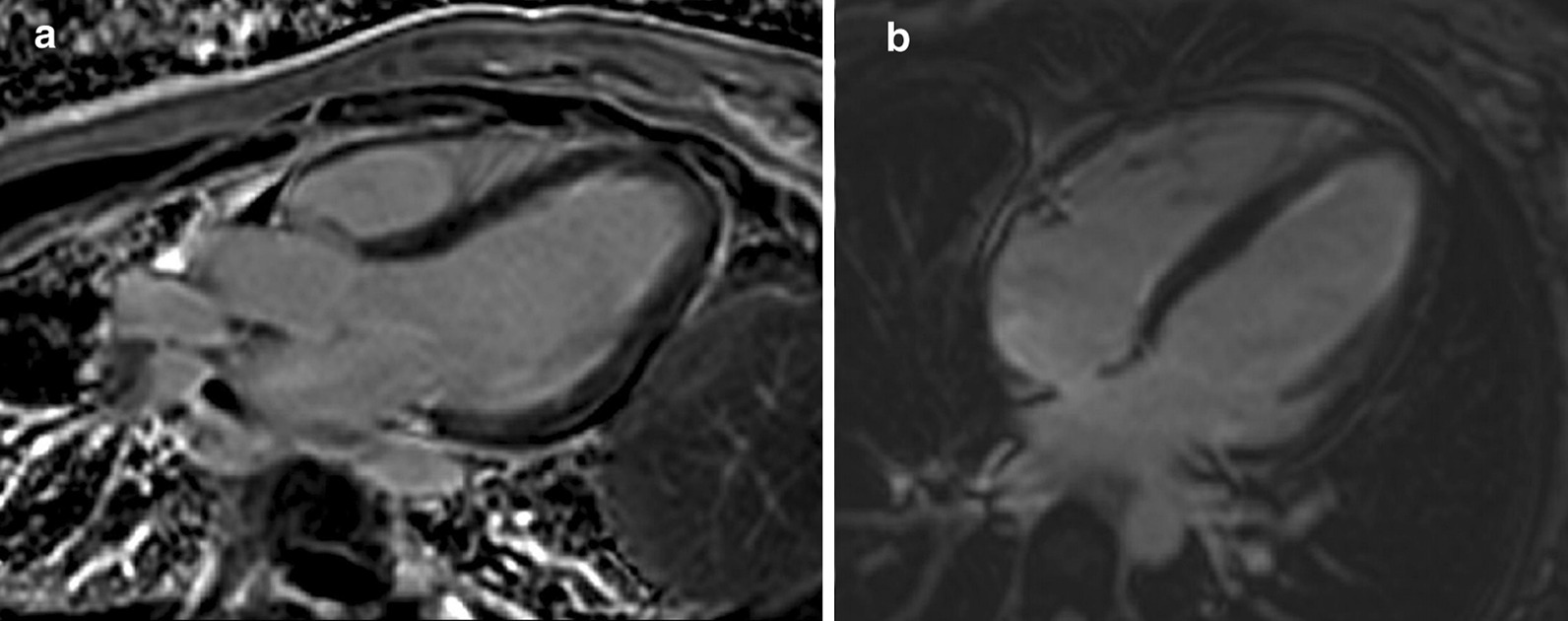
**Case 1.** Phase sensitive inversion recovery (PSIR). **a** Three chamber view with subtle subendocardial LGE of the mid and apical anteroseptal and inferolateral wall segments. **b** Four chamber view with subtle subendocardial LGE of the mid and apical inferoseptal and anterolateral wall segments

Late gadolinium enhancement (LGE) imaging using T1-weighted (T1w) imaging in short axis showed subendocardial enhancement in the mid to distal LV segments (Fig. 3). These findings correlated with phase sensitive inversion recovery (PSIR) LGE imaging in 3- and 4-chamber views (Fig. 4) also showing subtle subendocardial enhancement in the mid- and distal-LV segments.

**Fig. 5:**
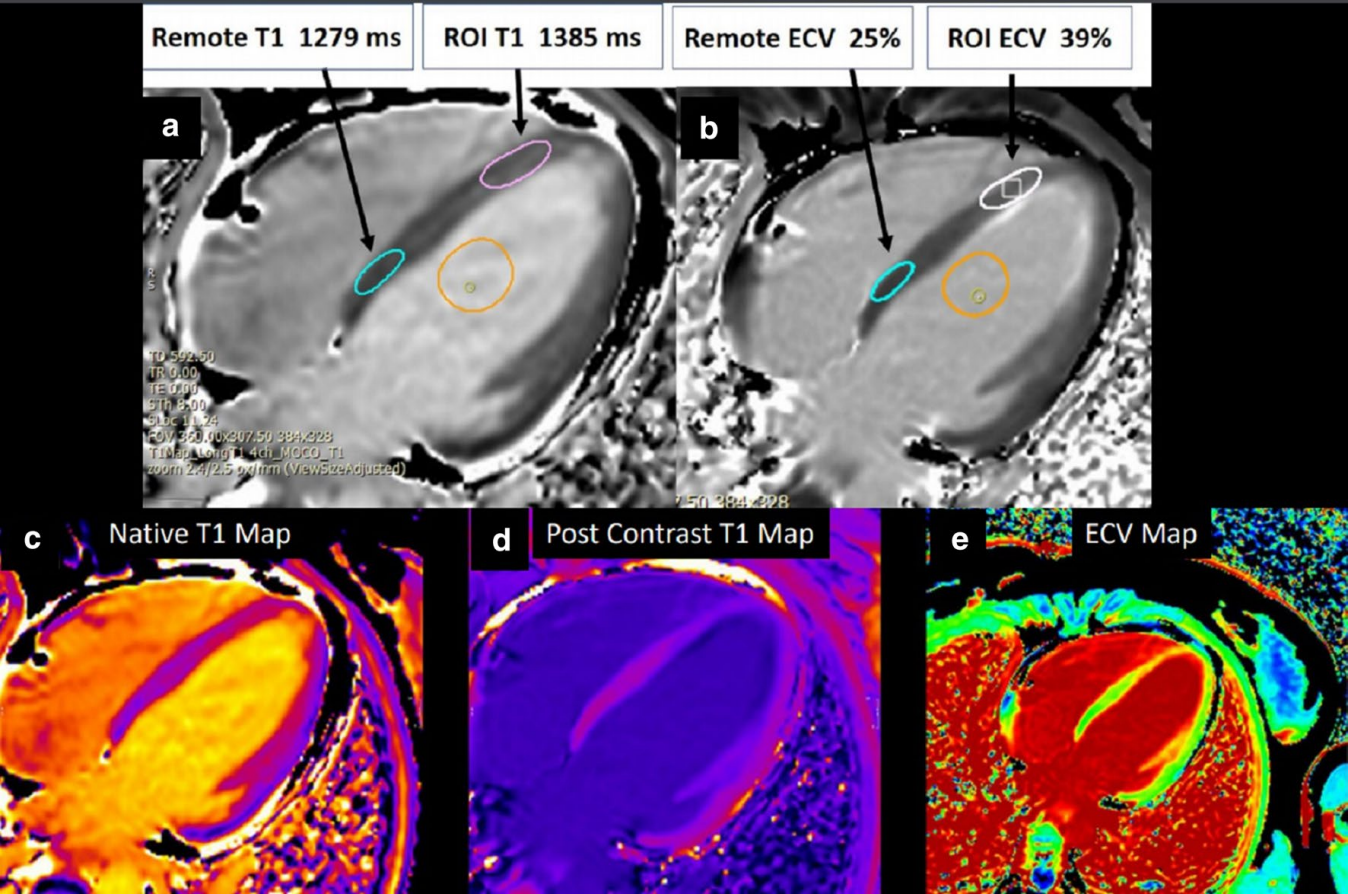
**Case 1.** Native T1, post-contrast T1, and extracellular volume (ECV) map in 4-chamber view. Native T1 (**a**) and ECV map (**b**) with abnormal measured native T1 and ECV in region of interest (apical septum) compared to remote region (basal septum). Pre-contrast T1 (**c**), post-contrast T1 (**d**), and ECV (**e**) map in 4-chamber view

Multi-parametric CMR: Pre- and post- contrast T1 mapping was performed a using modified Look-Locker inversion (MOLLI) sequence (Myomaps, Siemens Healthineers). Region of interest involving apical septum had an elevated T1 of 1385 ms (normal ~ 1150–1300 ms) as well as extracellular volume (ECV) of 39% (normal ~ 20–30%). In the remote myocardium (uninvolved basal inferoseptum) native T1 was in the normal range (1279 ms) and ECV was within normal limits (25%) (Fig. 5). Color maps confirmed subendocardial elevation in pre-contrast T1 and ECV in mid to apical LV segments (Fig. 5).

**Fig. 6:**
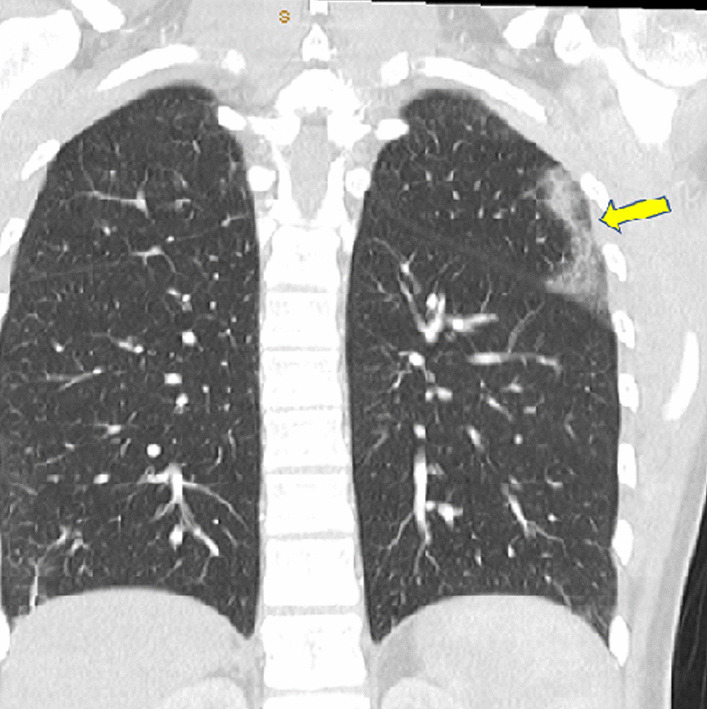
**Case 1.** Chest computed tomography (CT) coronal view. Nonspecific sub pleural pulmonary infiltrates in the left upper lobe (yellow arrow). CT guided needle biopsy of this lesion revealed eosinophilic infiltrates consistent with acute eosinophilic pneumonia

The patient was subsequently investigated for a systemic disease such as eosinophilic granulomatosis with polyangitis (EGPA). Chest computed tomography (CT) identified non-specific sub-pleural inflammatory changes (Fig. 6). CT guided needle biopsy of this lesion demonstrated eosinophilic infiltrates consistent with acute eosinophilic pneumonia with some vascular changes. The patient was subsequently treated with induction course of intravenous methyl prednisone followed by rituximab with good symptomatic and biochemical response to therapy (follow up ESR 4 mm/h, CRP of < 2 mg/l resolution of eosinophilia).

**Fig. 7:**
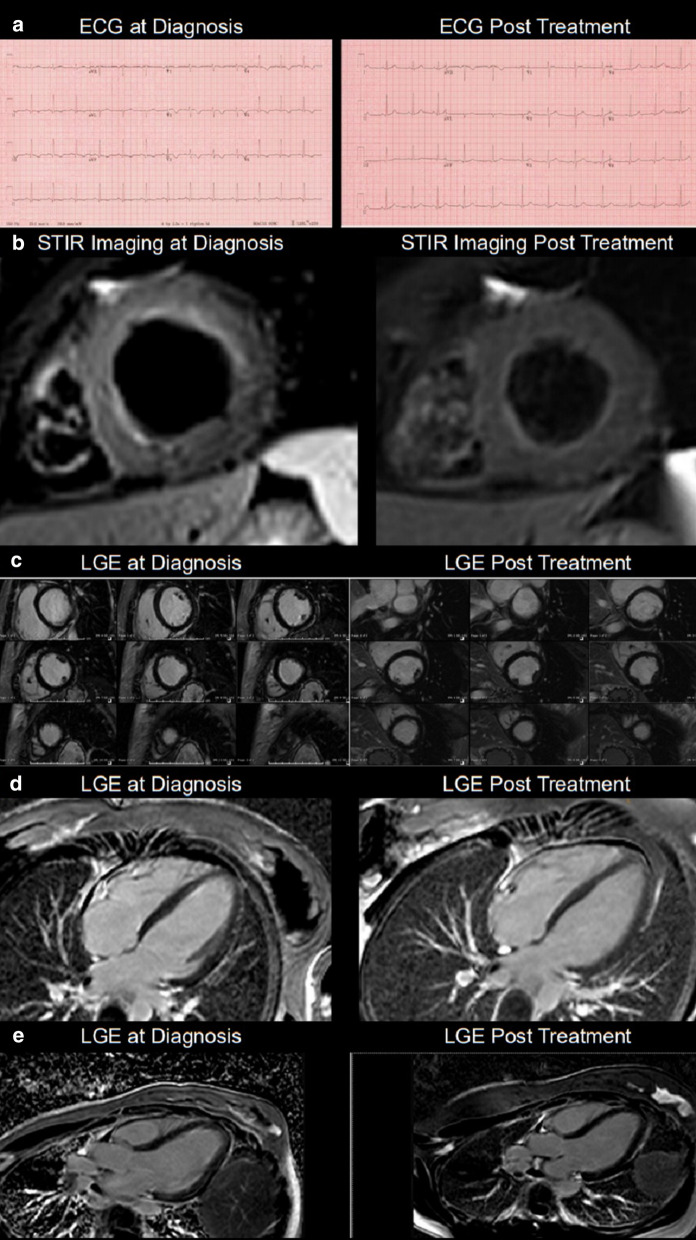
**Case 1.** Side by side comparison of pre- and post-treatment imaging. **a** 12 lead ECG. **b** T2-weighted STIR imaging LV short axis. **c** LGE short axis images. **d** GE 4 chamber image. **e** LGE 3 chamber image

Repeat CMR 6 months after treatment showed almost complete resolution of previously noted subendocardial abnormalities (Additional file 7: Movie S7, Additional file 8: Movie S8 and Fig. 7). Post treatment apical septum native T1 (1288 ms) and ECV (30%) returned to normal range.

### Conclusion

In the context of the patient’s history, the findings on CMR were suspicious for endo-myocarditis related to early stage EGPA. CT chest revealed clinically silent pulmonary infiltrates with CT-guided biopsy of these lungs infiltrates confirming eosinophilic pneumonia, and therefore avoiding cardiac biopsy. The final diagnosis was EGPA causing lateral tarsal artery thrombosis, with respiratory involvement, sinusitis and asymptomatic cardiac involvement. The patient improved with immune suppression. Repeat CMR performed 6 months post treatment revealed significant interval resolution of abnormal CMR findings.

### Perspective

EGPA, previously known as Churg-Strauss syndrome, is a multisystem disorder characterized by chronic rhinosinusitis, asthma, and prominent peripheral blood eosinophilia (≥ 1500 cells/microL and/or > 10% eosinophils on differential leukocyte count). The etiology of EGPA remains unknown. Asthma is the cardinal feature (occurring in more than 95% of patients) and usually precedes the vasculitic phase by approximately 8 to 10 years.

The American College of Rheumatology criteria for the classification of EGPA requires at least four of the following six criteria: asthma, eosinophilia > 10%, mononeuritis or polyneuritis, non-fixed pulmonary infiltrates, paranasal sinus abnormality and extravascular eosinophils [1]. It is a rare condition with an estimated annual incidence of 1.0 to 4.2 people per million [2].

Cardiac involvement is one of the more serious manifestations of EGPA, accounting for approximately half of attributable mortality. It should be suspected in the presence of refractory dyspnea, pericarditis related symptom, clinical evidence of heart failure (HF), or cardiac rhythm abnormalities, but can also be asymptomatic. Endomyocarditis may represent the most severe manifestation with eventually fatal outcome. A structured clinical assessment incorporating cardiac imaging with TTE and CMR can identify impaired cardiac function and endomyocardial abnormalities [3]. CMR is superior to TTE revealing cardiac abnormalities in 62% of Churg-Strauss patients compared to 50% by TTE [4]. Furthermore in this study, the absence of cardiac symptoms or ECG abnormalities did not exclude cardiac involvement, because CMR abnormalities could still be detected in 38% of these patients.

CMR is a useful tool for the differential diagnosis of HCM, as it can differentiate HCM from other mimicking conditions because of its superior spatial resolution and its ability to tissue characterize. Tissue characterization by CMR can differentiate eosinophilic endomyocarditis from apical HCM. Unlike apical HCM which usually has midmyocardial apical LGE, eosinophilic endomyocarditis typically has diffuse subendocardial enhancement, often associated with thrombus [5]. Furthermore this case highlights that CMR is more sensitive than TTE in detecting cardiac involvement in EGPA and occasionally CMR can provide important clues and contribute towards the EPGA diagnostic criteria. Finally, early diagnosis by CMR in this case led to early immunosuppressive treatment and potentially prevented catastrophic complications.

The CMR of Case 1 (Additional file CMR Link, https://www.precessionsaas.com/0757-1973-0753-0139/).

## Case 2: A case study in CMR based automated detection of left ventricular cardiotoxicity in a breast cancer patient after chemotherapy treatment

### Clinical history

**Fig. 8:**
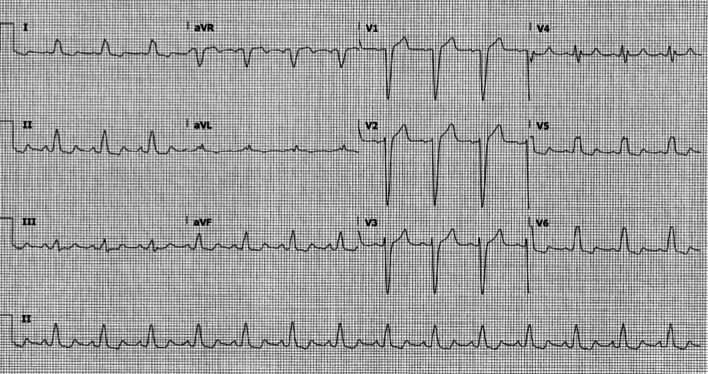
**Case 2.** Twelve lead ECG. Sinus rhythm with left bundle branch block

A 54 year old woman was diagnosed with left-sided breast cancer in October 2015. She was treated with adriamycin (cumulative dose of 400 mg/m^2^), cyclophosphamide and trastuzumab (Herceptin®, Genentech, South San Francisco, California, USA). She also received ADO–trastuzumab emtansine (Kadcyla®, Genentech) for a history of metastatic breast cancer previously receiving treatment with trastuzumab. During her chemotherapy regimen, a TTE revealed reduced LV systolic function (LVEF 48%). She was subsequently evaluated for the possibility of chemotherapy induced cardiomyopathy. Due to her decreased LVEF, continuous measurements of LVEF accompanied the trastuzumab treatment. Prior to chemotherapy, she had a past medical history of hypertension, left bundle branch block (LBBB) and hyperlipidemia. Medications included atorvastatin and carvedilol (12-lead ECG, Fig. 8). At this time the chemotherapy included carboplatin, gemcitabine, and trastuzumab. Given the decreased LVEF and existence of cardiovascular comorbidities, there was an immediate need to establish a diagnosis of cardiotoxicity.

### CMR findings

**Fig. 9:**
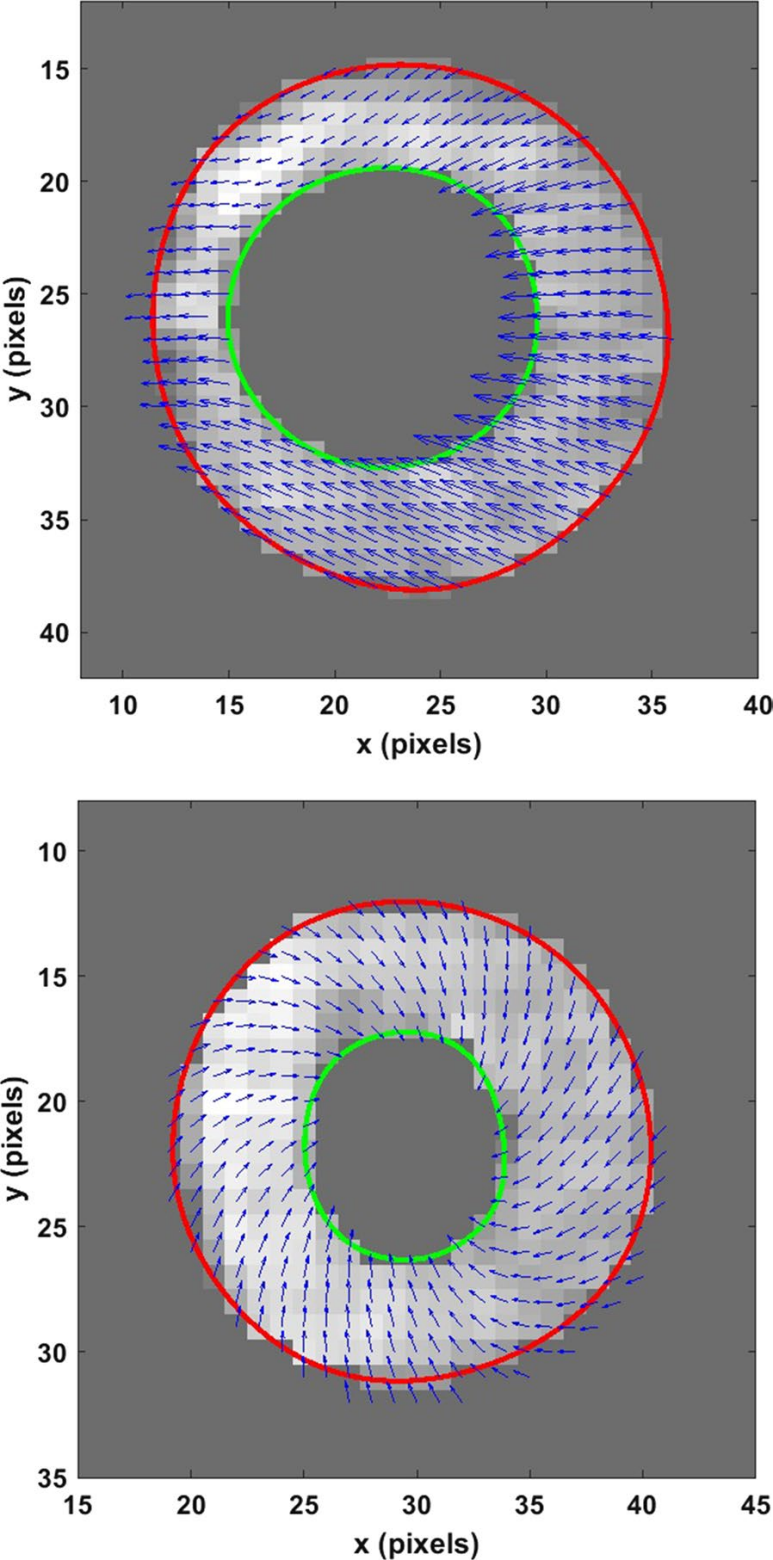
**Case 2.** Systolic displacement vectors in **a** the patient's and **b** a healthy subject’s mid-ventricle

**Fig. 10:**
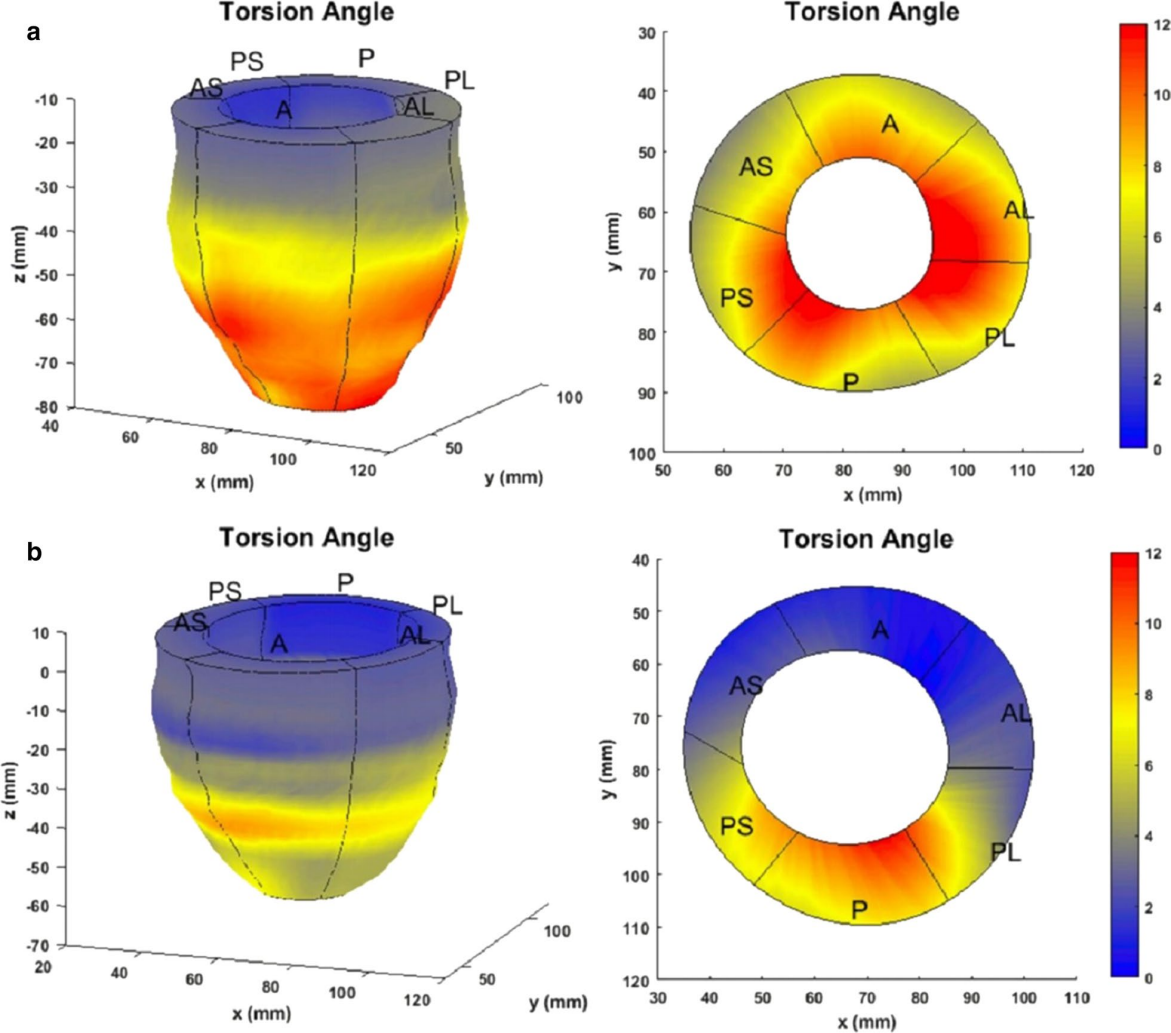
**Case 2.** Torsion in the LV and a mid-ventricular slice. **a** Healthy subject. **b** Abnormal torsion in the patient

A CMR with only myocardial strain analysis-based research investigation was carried out to further establish a diagnosis of cardiotoxicity. Traditional CMR sequences, including cine balanced steady state free precession (bSSFP), T1/T2 mapping, and LGE were not performed. Comprehensive analysis of her LV contractile parameters included CMR-based computations of 3D myocardial strains and torsion to predict the extent of dysfunction in a manner more quantitatively detailed than conventional LVEF measurements. CMR data were obtained with the Displacement Encoding with Stimulated Echoes (DENSE) sequence (Additional file 9: Movie S9) and 3D strains and torsion analysis conducted with an automated (patented) research algorithm consisting of multi-threshold image quantization for LV boundary detection, displacement analysis via phase unwrapping and strain analysis using a rapid meshfree methodology [6–10]. This research algorithm, which has been validated over several past studies, was used as a single-scan tool that provided comprehensive and automated analysis of LV chamber quantifications and 3D strain analysis. In this case the analysis required less than six minutes of processing time for 15 short axis slices [6–8]. Immediate signs of cardiac dysfunction can be observed from Fig. 9a where a biased and unusual septal twitch becomes obvious during systole compared to a normal systolic contraction in a healthy subject (Fig. 9b). The peak systolic longitudinal strain was significantly lower than healthier ranges found in our normal subjects’ database, which were − 9% (vs − 14 ± 4%), − 13% (vs − 19 ± 3%), − 14% (vs − 22 ± 4%) for the basal, mid-ventricular and apical LV regions, respectively. Similarly, peak systolic torsions were significantly different from the ranges found in the healthy subjects, which were 4.1 (vs 8.8 ± 1.7) radians for the mid-ventricular and 5.5 (vs 10.6 ± 1.6) for the apical LV regions (both with respect to the basal region). The lowered peak systolic torsions in comparison to a healthy subject can be seen in Fig. 10.

### Conclusion

Cardiotoxicity is a likely diagnosis given the patient’s age, chemotherapy exposure, and existence of cardiac comorbidities. Furthermore, follow up TTE now showed an LVEF < 25%. Abnormal CMR-based contractile function and strain analysis are strong indicators of cardiotoxicity that were not revealed by the earlier LVEF measurements. It also indicates the possibilities of consistent, long-term assessments of myocardial strain parameters supplementing routine cardiac examinations to support treatment options.

### Perspective

In regards to whether the findings of abnormal strain are related to the LBBB, a strong argument can be made that this is a combined deteriorating effect of cardiotoxicity and LBBB, where cardiotoxicity intensifies co-existing cardiac dysfunctions as shown in previous studies [11, 12]. The combined effect of two different dysfunctions is evident in this patient from the biased displacements (Fig. 9) found in the entire short axis cross section which is not exclusive to her septum. The existence of the cardiotoxicity is further supported by the reductions seen in global longitudinal strain (GLS) and torsion in the entire LV, which are parameters shown in previous studies as being effective in detecting cardiotoxicity [13, 14]. Importantly, the follow-up TTE showed an LVEF < 25%, a marked deterioration between studies. This conforms to guideline directed medical therapy (GDMT) based diagnosis of cardiotoxicity in breast cancer patients treated with anthracyclines and trastuzumab. Other CMR parameters of cardiotoxicity, T1/T2 contrast mapping showing the diffuse myocardial fibrosis (DMF) that occur with cardiotoxicity, could have mirrored the strain-based mechanisms of finding the underlying dysfunction [15]. However, the uncertainty remains that her LV dysfunction could entirely be an effect of LBBB. The newly patented algorithm for DENSE-based strain analysis used in this case is available via an application with user interface where automated analysis typically takes five minutes in a patient, validated against the gold standard of tissue tagging [6, 7, 10]. Hence, the DENSE strains are expected to be similar to those measured clinically with TTE and harmonic phase (HARP), and HARP protocols are commonly validated in comparison to tissue tagging. Echocardiogram strain measurements as recommended by the American Society of Echocardiography (ASE) for post-chemotherapy assessments (including associated dose adjustment) would have added more robustness to this case [16]. DENSE is best suited for imaging systolic function in cardiotoxicity as it is fast with high resolution unlike CMR tissue tagging. DENSE does not suffer from excessive artifacts, noise, or decreased resolution due to the asymmetrically sampled spectral peak also found in HARP. The repeatability and reproducibility of DENSE data is independent of operator accuracy, a common challenge in echocardiographic strain [6]. Ultimately, many breast cancer patients who have been cured via chemotherapy may suffer from progressive LV dysfunction due to chemotherapy induced cardiotoxicity and symptomatic HF [12–15, 17]. These studies repeatedly emphasize that despite recent advancements in HF treatment, a majority of patients with cardiotoxicity-induced cardiac dysfunction show no improvement in LV systolic function, and that the benefits of detecting cardiotoxicity early in its manifestation (not always apparent from small declines in LVEF) cannot be ignored.

A study by Siedman et al. demonstrated that the abnormal septal motion in systole undermined treatment efficacy when patients were administered a combined dose of chemotherapy agents including anthracyclines, trastuzumab and cyclophosphamides [17]. In this context, studies also show that myocardial dysfunction measured with global and regional computations of 3D strain (and torsion) are more sensitive toward early detection of cardiotoxicity prior to LVEF deterioration. Additionally, the ability to detect cardiotoxicity more sensitively via analysis of strain parameters (longitudinal strain and torsion) was shown by two previous studies [13, 14]. Hence, detecting cardiotoxicity related dysfunction via CMR DENSE-based strain analysis is a viable methodology that should be practiced more in mainstream clinical diagnosis of chemotherapy induced cardiomyopathy toward further improving the quality of surveillance required in this patient population.

The CMR of Case 2 (Additional file CMR Link, https://www.cloudcmr.com/3357-1973-2158-0172/).

## Case 3: Left ventricular thrombus or myxoma: the use of multimodality imaging

### Clinical history

A 46 year old woman presented with generalized abdominal pain. Initial evaluation with abdomen/pelvis CT demonstrated wedge shaped areas of low attenuation in her spleen and right kidney consistent with infarction. She also had a dynamic ileus secondary to bowel infarction along with findings suggestive of an embolus in her superior mesenteric artery (SMA). CT also revealed a possible 2 × 4.1 cm “mass” within the LV. Empiric anticoagulation with heparin was started and she underwent urgent exploratory laparotomy, small bowel resection, SMA embolic material removal and patch angioplasty of the SMA.

**Fig. 11:**
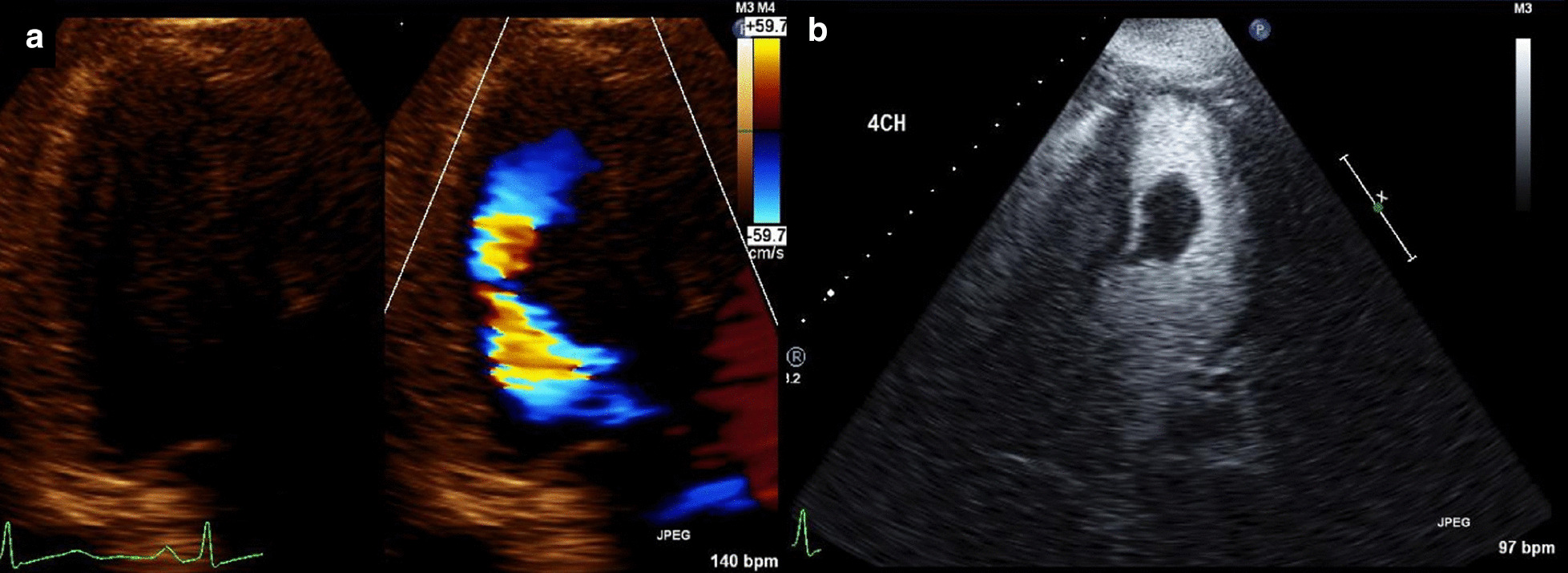
**Case 3.** Transthoracic echocardiography (TTE) apical LV views with and without ultrasound enhancing agent

**Fig. 12:**
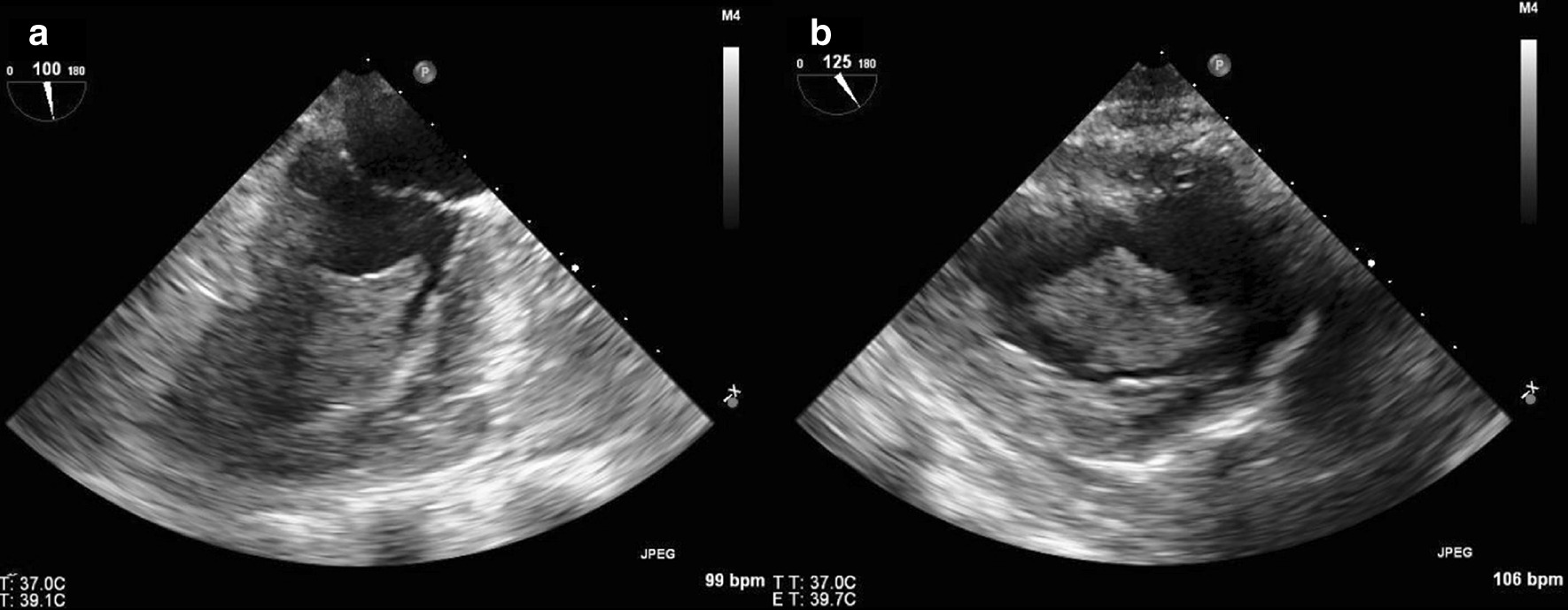
**Case 3.** Transesophageal echocardiographic (TEE) mid-esophageal 2 chamber and transgastric projections

TTE on the day after admission showed an LVEF of 65% without regional dysfunction. It also confirmed an LV “mass” near the outflow tract which measured 3.2 × 2.3 cm (Fig. 11). The mass was hypovascular and pedunculated with an attachment to the basal anteroseptal wall. Second look surgery on the following day showed that the areas of questionable viability from her first surgery were frankly ischemic, as a result of which she underwent ileostomy and jejunostomy, leaving only 20 cm of viable terminal ileum and the entire colon. She underwent a transesophageal echocardiogram (TEE) 2 days later, which corroborated the TTE findings, revealing an LV “mass” (Fig. 12).

**Fig. 13:**
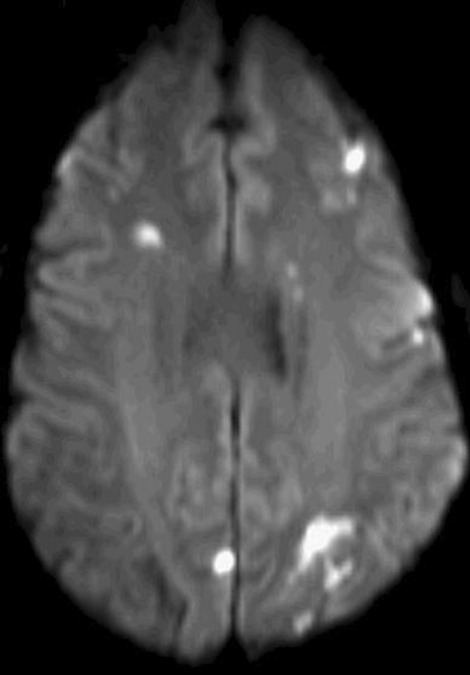
**Case 3.** Diffusion weighted brain MRI showing multifocal infarctions consistent with embolism

Initial interpretation of the “mass” favored a myxoma but a thrombus was also suspected. A CMR performed a week after her admission showed findings consistent with a thrombus that had embolized from the LV into the aortic arch. On the following day a brain magnetic resonance imaging (MRI) revealed multiple cerebral infarctions in her internal carotid artery territory (Fig. 13). Pathology results confirmed that the SMA embolic material was indeed thrombus. Due to difficulty managing her heparin, anticoagulation was changed to enoxaparin. The patient was found to suffer from left middle cerebral and posterior cerebral artery strokes when she had symptoms of right hemiparesis, dysarthria and word finding difficulty. Thrombolysis or thrombectomy were not undertaken as the patient was anticoagulated and had poor carotid access. She had residual right side hemianopsia with no other residual deficits.

Her anticoagulation was then changed to fondaparinux, to be continued indefinitely. She was ultimately discharged to inpatient rehabilitation with plans to be evaluated later for intestinal transplant due to short gut syndrome. After rehabilitation, she was able to walk but used a wheelchair and walker for ambulating a longer distance.

### CMR findings

The CMR revealed a LVEF of 60% with normal dimensions and regional systolic function without any LV mass (Additional file 10: Movie S10, Additional file 11: Movie S11, Additional file 12: Movie S12, Additional file 13: Movie S13).

The patient had a bovine aortic arch anatomy and was found to have a 2.8 × 1.9 cm occlusive thrombus in the proximal aortic arch extending into the proximal innominate artery. The thrombus had low signal intensity on bSSFP imaging, short T1 and T2 consistent with methemoglobin, and was hypointense on LGE (Additional file 14: Movie S14, Additional file 15: Movie S15, Additional file 16: Movie S16, Additional file 17: Movie S17, Additional file 18: Movie S18, Additional file 19: Movie S19).

### Conclusion

We report a case of an LV thrombus that embolized in its entirety to the aortic arch, innominate artery and caused multi focal infarcts in the SMA and internal carotid artery distributions. Our report emphasizes the use of comprehensive CMR imaging in the assessment and differentiation of cardiac masses. In our patient, even though the “mass” had embolized by the time the CMR was performed, its tissue characterization abilities were critical in identifying that it was a thrombus. The mass had a high signal and a short T1 consistent with methemoglobin. Overall, due to a larger field of view, greater spatial resolution, lack of attenuation, and ability to image in any plane along with its superior tissue characterization properties, CMR is the gold standard for assessment of cardiac masses [23].

### Perspective

There have been several case reports of thrombus mimicking cardiac tumors. LV thrombus formation is a well-known complication of a myocardial infarction, LV aneurysm, cardiomyopathy or a hypercoagulable state. It is uncommon for a thrombus to form in a normal LV in the absence of a wall motion abnormality. Initial interpretation of the “mass” favored myxoma due to the absence of these factors. The specific etiology for formation of thrombus is unclear as her imaging and hypercoagulable work up did not point to a specific cause. Notably, she was on medroxyprogesterone injections but her hypercoagulable work up was negative for anticardiolipin antibodies, antiphospholipid antibodies, lipoprotein A, beta 2 glycoprotein, and JAK (Janus Kinsae) 2 mutation.

Imaging plays a critical role in establishing a diagnosis and planning management. TTE, TEE, and CMR have all been used to detect the presence of cardiac tumors and thrombi [18]. CMR has a superior sensitivity and specificity of 88 ± 9% and 99 ± 2% in comparison to echocardiography and has enhanced ability to identify thrombi and differentiate from other cardiac masses because of superior tissue characterization [19]. T1 weighted CMR imaging has been successful in identifying acute/subacute thrombi due to the short T1 effect of methemoglobin [20, 21]. On CMR, recent thrombi have a shorter T1 than old thrombi due to the presence of methemoglobin and hence a brighter signal on T1 weighted images [22].

It is important to establish whether the mass is a tumor, thrombus or vegetation due to their different complications and therapeutic implications. Treatment of thrombi includes adequate anticoagulation. In our patient, no intervention was performed as the thrombus had already embolized into the aortic arch.

The CMR of Case 3 (Additional file CMR Link, https://www.cloudcmr.com/1518-5003-5038-1960/).

## Case 4: Hypovascular renal cell carcinoma infiltrating the left atrium through the pulmonary veins

### Clinical history

A 43-year-old man underwent left side radical nephrectomy four months previously for removal of a biopsy proven clear renal cell carcinoma. The patient did not receive chemotherapy. The patient presented with complaints of shortness of breath and hemoptysis. TTE revealed a left atrial (LA) mass with no right heart involvement detected. As renal cell carcinomas most commonly involve the heart by extension through the inferior vena cava (IVC) and right-side chambers, a LA thrombus was initially suspected in this case. The patient was referred for CMR to verify the diagnosis prior to starting anticoagulants.

### CMR findings

CMR was performed using at 1.5 T (Magnetom Aera, Siemens Healthineers). Given the patient’s shortness of breath, real time axial cine and black blood axial images were acquired through the chest and upper abdomen. These images revealed widespread metastatic masses involving the liver, mediastinum and lungs with extension through the pulmonary veins (Additional file 20: Movie S20). The mediastinal masses appeared continuous with the LA mass, raising the possibility of extension of the malignant tumor to the LA.

For mass characterization, ECG gated axial T2, axial T2 with fat suppression, axial T1, and first pass contrast imaging after injection of a gadolinium-based contrast agent (Magnevist, Schering AG, Berlin, Germany, 0.2 mmol/kg), 1, 5 and 10 min after contrast injection images were acquired. ECG gated delayed post contrast images were acquired with high inversion time (700 ms) to assess for possible thrombus.

**Fig. 14:**
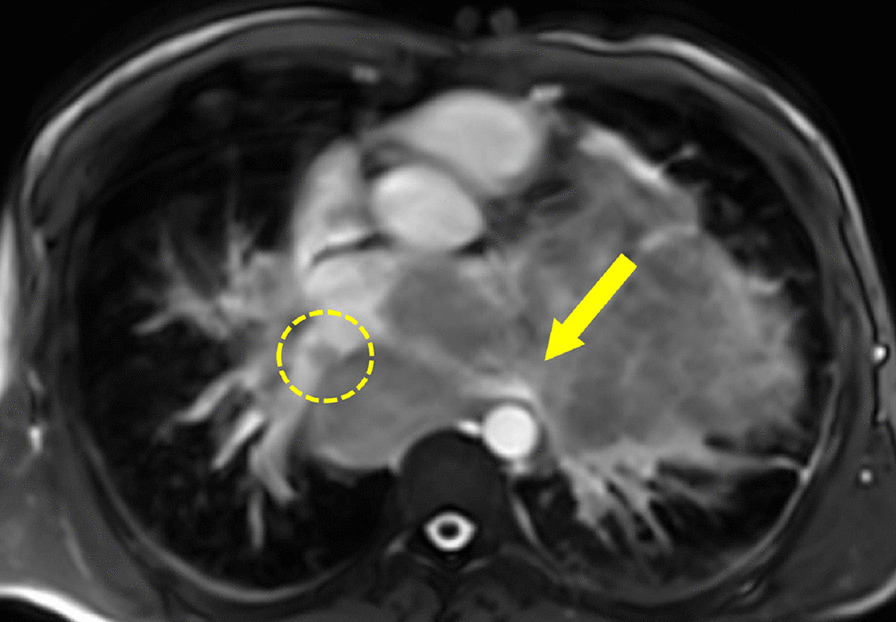
**Case 4.** Cine balanced steady state free precession (bSSFP) image shows the right pulmonary veins (yellow circle) and left pulmonary veins (yellow arrow) infiltrated by the mediastinal mass

A single, well defined, infiltrative mass was seen in the LA measuring 5.7 × 5.3 × 3.6 cm (Additional file 21: Movie S21, Additional file 22: Movie S22, Additional file 23: Movie S23). The LA mass compromised mitral valve inflow, protruding into the LV. No other cardiac chamber or valvular involvement was detected. The mass extended into the LA through infiltration of the left sided pulmonary veins, a part of multiple mediastinal and bilateral lung metastases (Fig. 14).

**Fig. 15:**
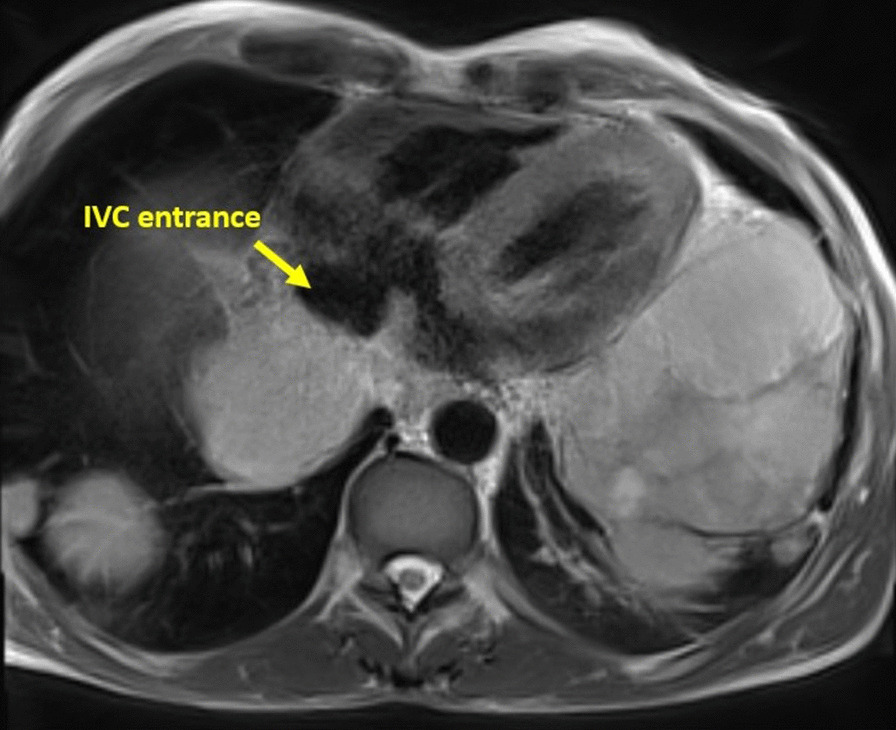
**Case 4.** Axial T2 fat suppression image. Pericardial infiltration over the atria, yet, no effusion and no atrial extension. The inferior vena cava (IVC) entrance to the right atrium is spared

**Fig. 16:**
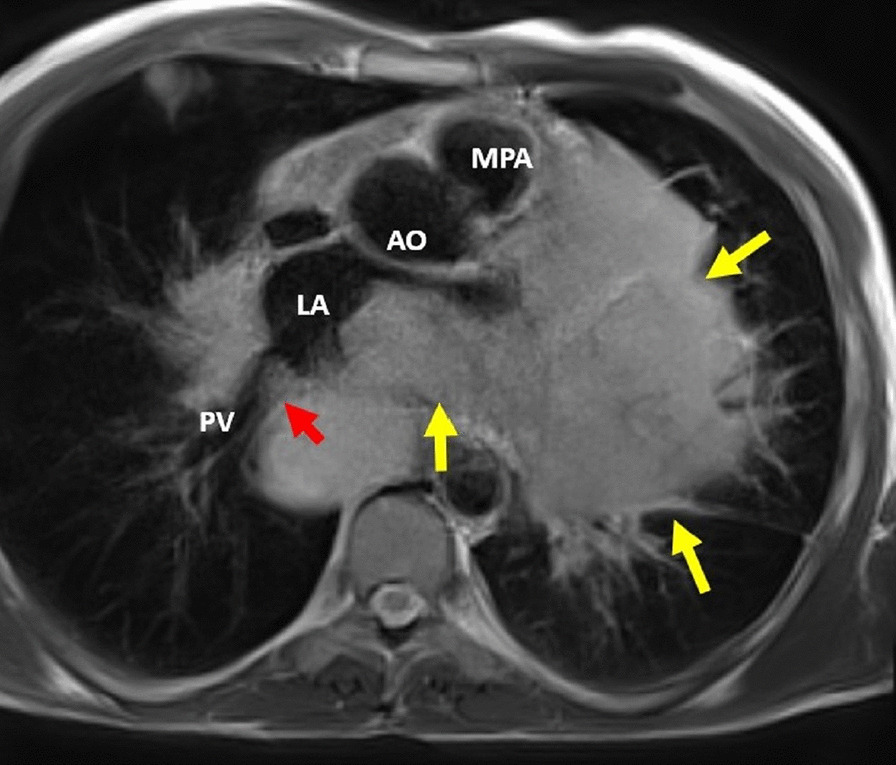
**Case 4.** Axial T2 image. Large mediastinal metastatic mass (yellow arrows) infiltrating the left atrium (LA) and encasing the main pulmonary artery (MPA). The mass appears separable from the ascending aorta (AO). The right lower pulmonary vein (PV) appears infiltrated by mass (red arrow)

**Fig. 17:**
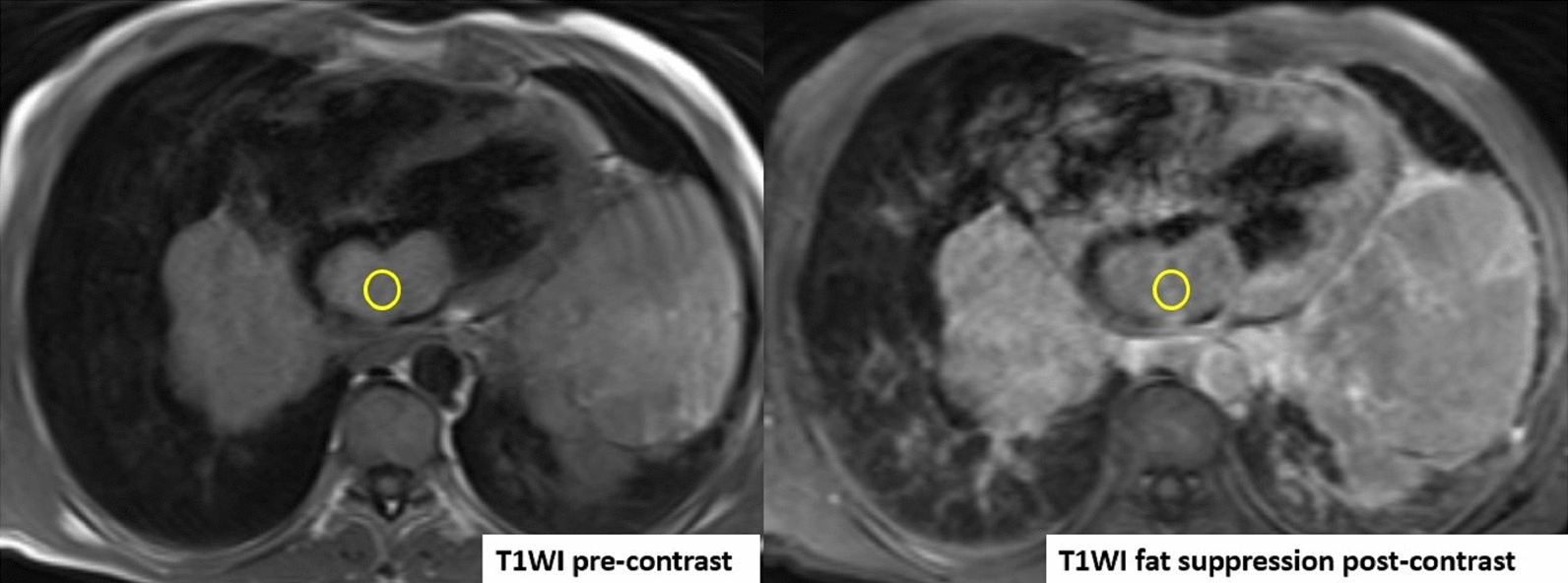
**Case 4.** Axial T1 image before and after contrast. Increased signal intensity in the post-contrast study relative to the pre-contrast study

Biventricular size and regional/global systolic function were normal. Focal pericardial infiltration over the LV and along the posterior and inferior pericardial surfaces was noted (Fig. 15). Multiple pleural masses were also seen. No pericardial or pleural effusions were present. The mass demonstrated hyperintense T1 and T2 signal relative to the myocardium (Figs. 16, 17).

**Fig. 18:**
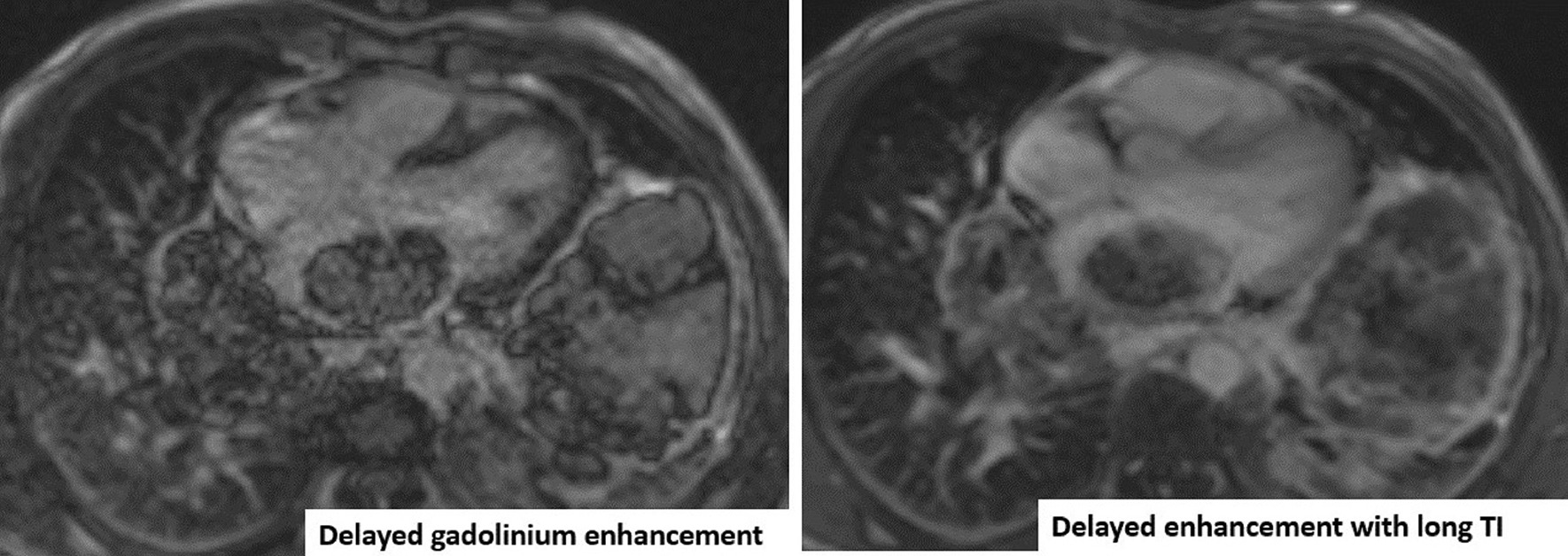
**Case 4.** LGE with myocardial nulling and long inversion time. In comparison to the long TI image, there is still enhancement in the mass with no jet-black signal seen in the long TI image

Mild enhancement was noted during first pass perfusion (Additional file 24: Movie S24), with mild enhancement also seen in early post contrast images. This could be secondary to contrast moving over the surface of the mass as opposed to true mass perfusion. Decreased LGE was detected (Fig. 18). There was a possible hemorrhagic component. No calcifications, central breakdown, or fatty components to the mass were detected.

Regarding the great vessels, the thoracic aorta was spared from metastatic involvement, with the arch and descending thoracic aorta passing tangential to the mediastinal masses with no definite intravascular extension or narrowing of the arch detected. The left pulmonary artery is infiltrated with attenuation of its diameter. No definite intravascular involvement was detected in the main or right pulmonary arteries (Additional file 25: Movie S25).

**Fig. 19:**
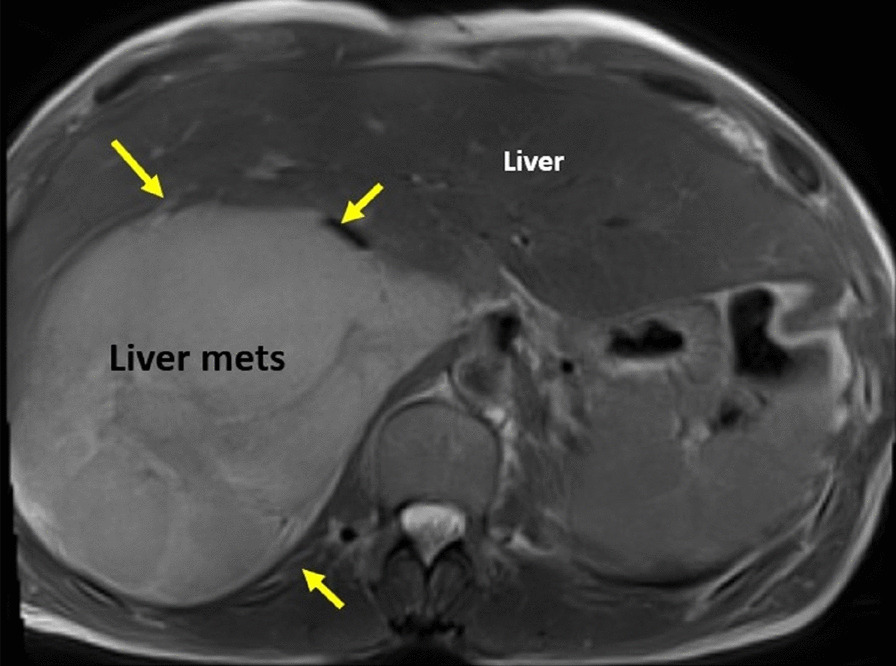
**Case 4.** Large liver mass indicated by yellow arrows

There is a very large liver mass measuring 14 × 11 cm seen infiltrating the right sided hepatic veins and compressing the hepatic portion of IVC (Fig. 19). There is absent connections between the extra-thoracic and hepatic vein malignant tissue and the intra-cardiac tumor. While the hepatic portion of the IVC was spared, wall infiltration with narrowing was seen just before entrance into the right atrium (RA).

Multiple metastases were seen in the lungs bilaterally, with largest lesion seen in the left lower lung lobe measuring 10 × 7.5 cm. Multiple mediastinal and para-hilar lymph nodes were seen, the largest of which was in the subcarinal group measuring 4.7 cm.

### Conclusion

While aggressive chemotherapy and thrombolytics were initially planned, the plan was altered due to the CMR findings. Based on the CMR findings, the masses visualized were felt to most likely be consistent with widespread metastatic disease. Ultimately, palliative chemotherapy was offered and the patient unfortunately died. Pathology assessment of the cardiac and mediastinal masses was not performed.

This case highlights the importance of an organized approach regarding clinical decision making and diagnostic imaging studies performed, as management approaches can sometimes be changed based on confirmatory testing.

### Perspective

CMR is an invaluable noninvasive imaging modality to assess cardiac masses. Table 1 provides a summary of the CMR imaging characteristics seen with various types of cardiac masses [24].

**Table 1:** CMR imaging characteristics of various types of cardiac masses

Tumor	Common location	T1 weighted	T2 weighted	T1 post contrast enhancement	Late gadolinium enhancement	Fat suppression
Pseudotumors						
Thrombus	Atrial appendage in valve disease and/or atrial fibrillation; Ventricles related with aneurysm or myocardial infarction	Hypointense (hyperintense if acute or subacute)	Hypointense (hyperintense if acute)	No	Hypointense with long TI time	No change
Pleuro-pericardial cyst	Right cardiophrenic angle is the most common; can be anywhere in mediastinum	Hypointense; can be hyperintense if hemorrhagic	Hyperintense	No	Hypointense	No change
Vegetation	Cardiac valves and aortic grafts	Frequently not visible	May be visualized if large	Can enhance	Can enhance (if wide based and large	No change
Benign Cardiac Tumors						
Myxoma	Left atrium Fossa ovalis	Variable	Variable	Yes	Heterogeneous	No change
Lipoma	Any chamber, intramyocardial or intracavitary	Hyperintense	Hyperintense	No	Hypointense	Hypointense (signal dropout)
Papillary fibroelastoma	cardiac valves	Isointense	Hyperintense	Yes	Hyperintense	No change
Rhabdomyoma	Any chamber, intramyocardial or intracavitary	Isointense	Hyperintense	Yes	Isointense	No change
Fibroma	Intramyocardial (ventricular septum or free wall)	Isointense	Hypointense	Hypointense core; Isointense periphery	Hyperintense	No change
Hemangioma	Ventricles intramyocardial / intracavitary	Intermediate	Hyperintense	Yes	Variable	No change
Malignant Cardiac Tumors						
Angiosarcoma	Right atrium, pericardial and myocardial invasion	Heterogeneous	Heterogeneous	Heterogeneous	Heterogeneous	No change
Rhabdomyosarcoma	Any chamber; pericardial or pleural involvement	Intermediate	Hyperintense	Variable	Heterogeneous	No change
Lymphoma	Pericardial effusion; Intracardiac masses (mostly involves the right heart)	Isointense	Isointense to Hyperintense	Variable	Variable	No change
Metastases	Multiple lesions, mostly involves the right heart, pericardial effusion	Hypointense	Hyperintense	Variable	Heterogeneous	No change

Renal cell carcinoma patients remain at risk for metastatic disease for many years after diagnosis, with the clear cell type having the highest risk for metastatic spread [25]. Cardiac metastasis of renal cell carcinoma is an exceptional event, particularly when there is lack of IVC involvement [26].

To our knowledge, only 14 cases have been reported worldwide involving the pulmonary veins and or the LA without IVC infiltration [27–29]. Absent extra-renal metastatic spread before radical nephrectomy, absent IVC infiltration, and atypical route of metastases all were unusual findings in this case.

The CMR of Case 4 (Additional file CMR Link, https://www.cloudcmr.com/1857-1973-2528-0116/).

## Case 5: Neonatal giant atrial hemangioma—a rare cardiac tumor

### Clinical history

A 30 year old gravida 2 para 1 female was referred for evaluation of a fetal intracardiac mass noted on routine obstetric ultrasound. Fetal echocardiogram at 26-weeks’ gestation revealed a single, large, homogenous, hyperechoic cardiac mass occupying bi-atrial cavities with unobstructed intra-cardiac flows (Additional file 26: Movie S26, Additional file 27: Movie S27). There was normal biventricular systolic function and no pericardial effusion. Serial fetal echocardiograms showed the size of the mass increasing in proportion to the rest of the cardiac size.

Postnatally, the infant was clinically well. TTE confirmed a large mass measuring 11 × 13 mm, arising from the Eustachian valve and predominantly occupying the RA cavity, with unobstructed intra-cardiac flows (Additional file 28: Movie S28, Additional file 29: Movie S29, Additional file 30: Movie S30, Additional file 31: Movie S31, Additional file 32: Movie S32). There was a patent foramen ovale with left to right shunt.

Low dose aspirin was started for thromboprophylaxis. Tuberous sclerosis work-up including a brain MRI, renal ultrasound and genetic testing were negative. Patient was discharged home with plan for outpatient CMR. The infant continued to thrive well with no complications related to the mass.

### CMR findings

CMR was performed to characterize the tumor. In view of patient age, the procedure was performed under general anesthesia with breath-holding. On cine bSSFP sequences, there was an irregular dumbbell-shaped mass attached to the Eustachian valve on the right side of the interatrial septum measuring 5 × 11 cm (axial plane) and 8 × 12 mm (sagittal plane) (Additional file 31: Movie S31). There was no obstruction to intracardiac flows.

**Fig. 20:**
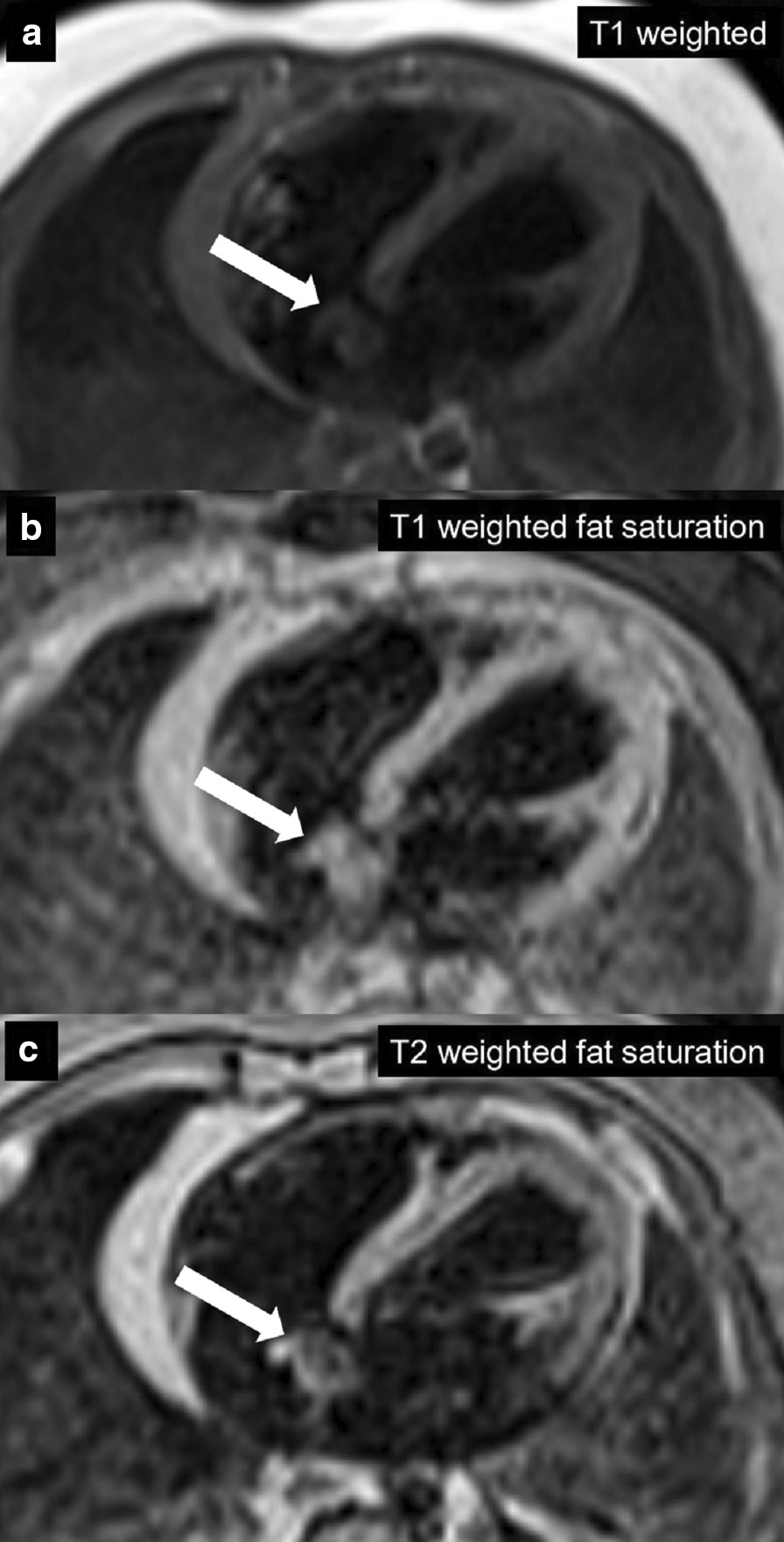
**Case 5.** T1 weighted with and without fat saturation and T2 weighted with fat saturation axial images. The atrial mass (white arrow) was isointense on T1w sequences (**a**, **b**) and hyperintense on T2w sequences (**c**)

**Fig. 21:**
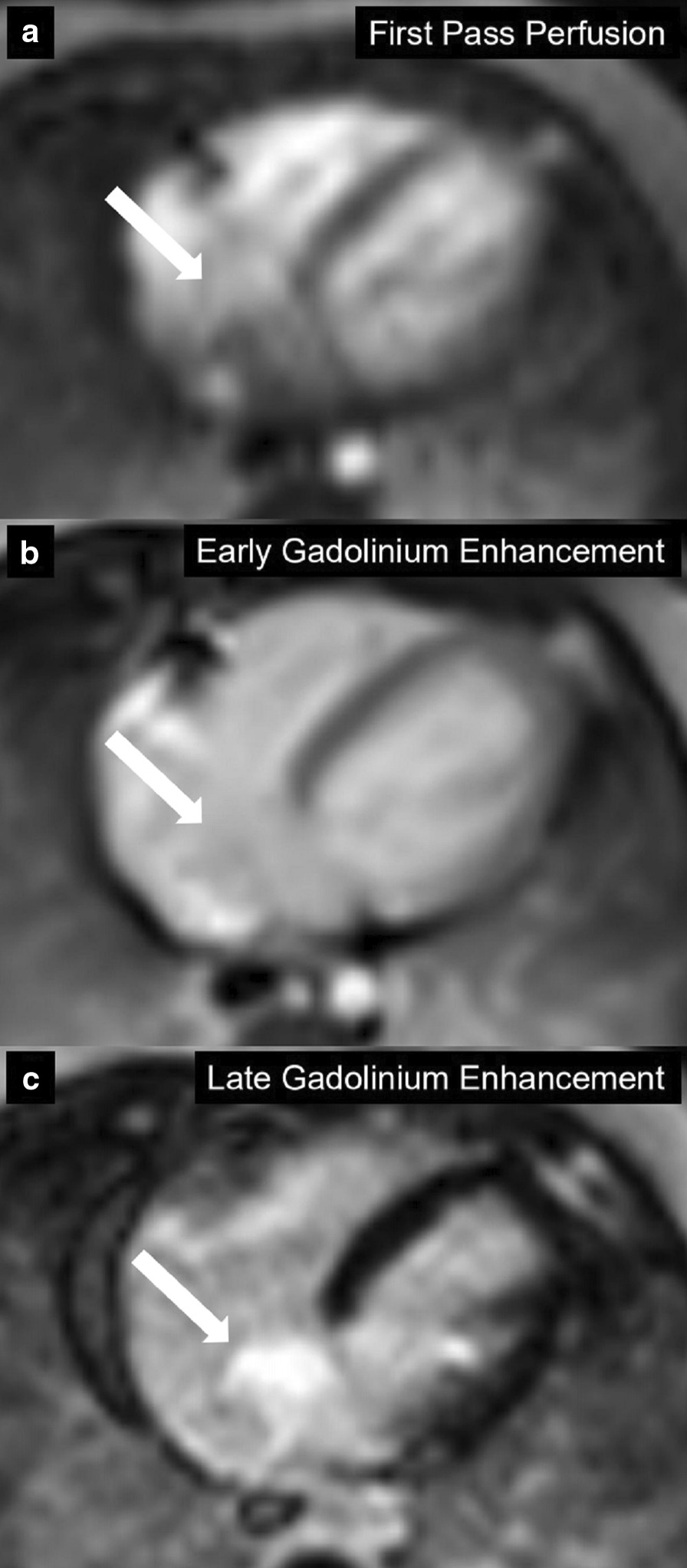
**Case 5.** Axial first pass perfusion, early gadolinium enhancement, and LGE images. The atrial mass (white arrow) has contrast uptake on first pass perfusion (**a**), not well seen on early enhancement (**b**), and hyperintense on LGE (**c**)

The mass was isointense on T1w sequences with and without fat saturation, suggesting a non-fatty mass. On T2w sequence with fat suppression the mass was non-homogenously hyperintense (Fig. 20). There was early uptake of contrast to the center of the mass on first pass perfusion suggesting a vascular tumor (Additional file 32: Movie S32). The mass was not visualized on early gadolinium sequences (TI 600 ms) while there was intense enhancement on LGE imaging (Fig. 21).

### Conclusion

In view of the patient’s age, tumor location, and clinical presentation, a benign hemangioma was considered the most likely diagnosis based upon the current CMR knowledge (see Table 2) [30]. With a mass that was not growing in size and no clinical complications in the form of arrhythmia, heart failure or obstruction, there was no indication to obtain a tissue diagnosis. The infant was conservatively managed with low dose aspirin and was closely monitored with serial TTEs that revealed decreasing tumor size.

**Table 2:** − , iso- or hypointense; +, hyperintense; Fat sat, fat saturation; FPP, first pass myocardial perfusion; LGE, late gadolinium enhancement; bSSFP, balanced steady state free precession

Tumor type	Location	SSFP	T1w	T1w + Fat sat	T2w	FFP	LGE
Vascular	Variable	Variable	–	–	+ (variable)	Strong	+ (variable and heterogenous)

### Perspective

CMR sequences currently available do not allow distinction among benign vascular tumors (e.g., hemangioma), malignant vascular tumors (e.g., angiosarcoma), vascular malformations, and tumors with ample vascular supply (e.g., paraganglioma). Primary vascular cardiac tumors such as hemangiomas are extremely rare (< 10% of all primary cardiac tumors in children). Congenital cardiac hemangiomas can present as fetal arrhythmia, hydrops fetalis and intrauterine fetal demise. Neonates can be asymptomatic, but can also present with cardiac tamponade, arrhythmias, congestive heart failure or sudden death [31].

Asymptomatic children are best managed conservatively by closely monitoring for development of complications. The role of medical therapy is limited; there is no proven role for beta blockers, while steroids have mixed results. Surgery is only offered for rapidly growing or symptomatic tumors and, steroid therapy is reserved for patients with unresectable tumors [32]. The present case highlights the role of CMR in non-invasive diagnosis of vascular cardiac tumors by a combination of imaging sequences for tissue characterization [33].

The CMR of Case 5 (Additional file CMR Link, https://www.precessionsaas.com/3657-1973-9453-0171/).

## Case 6: The role of modality imaging in evaluation of new onset ventricular tachycardia.

### Clinical history

**Fig. 22:**
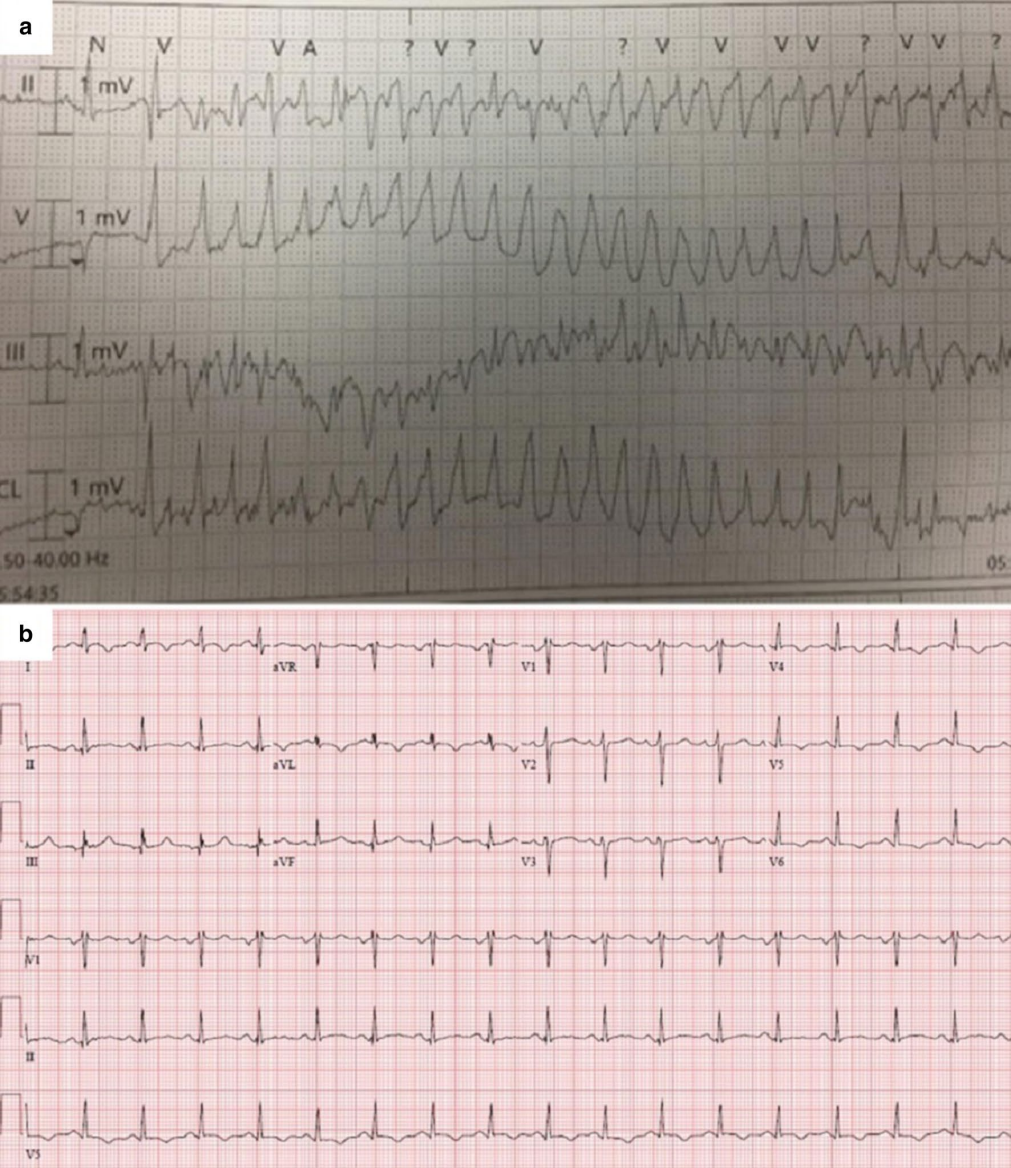
**Case 6.** Twelve lead ECG. **a** Ventricular tachycardia on presentation. **b** Sinus tachycardia with T wave inversion of lateral leads

A 56 year old male with a past medical history of antiphospholipid syndrome, chronic kidney disease stage III (resulting from a renal infarct from an embolic thrombus), and essential hypertension presented to the emergency department after a near syncopal episode while he was uploading boxes. Upon emergency medical services (EMS) arrival, he was noted to be in monomorphic ventricular tachycardia (VT) (Fig. 22) that needed immediate synchronized cardioversion with subsequent resolution of his symptoms. Upon arrival to the emergency room, the patient was hemodynamically stable with benign cardiovascular examination. Resting ECG (Fig. 22) showed sinus tachycardia with T wave inversions over the lateral leads.

**Fig. 23:**
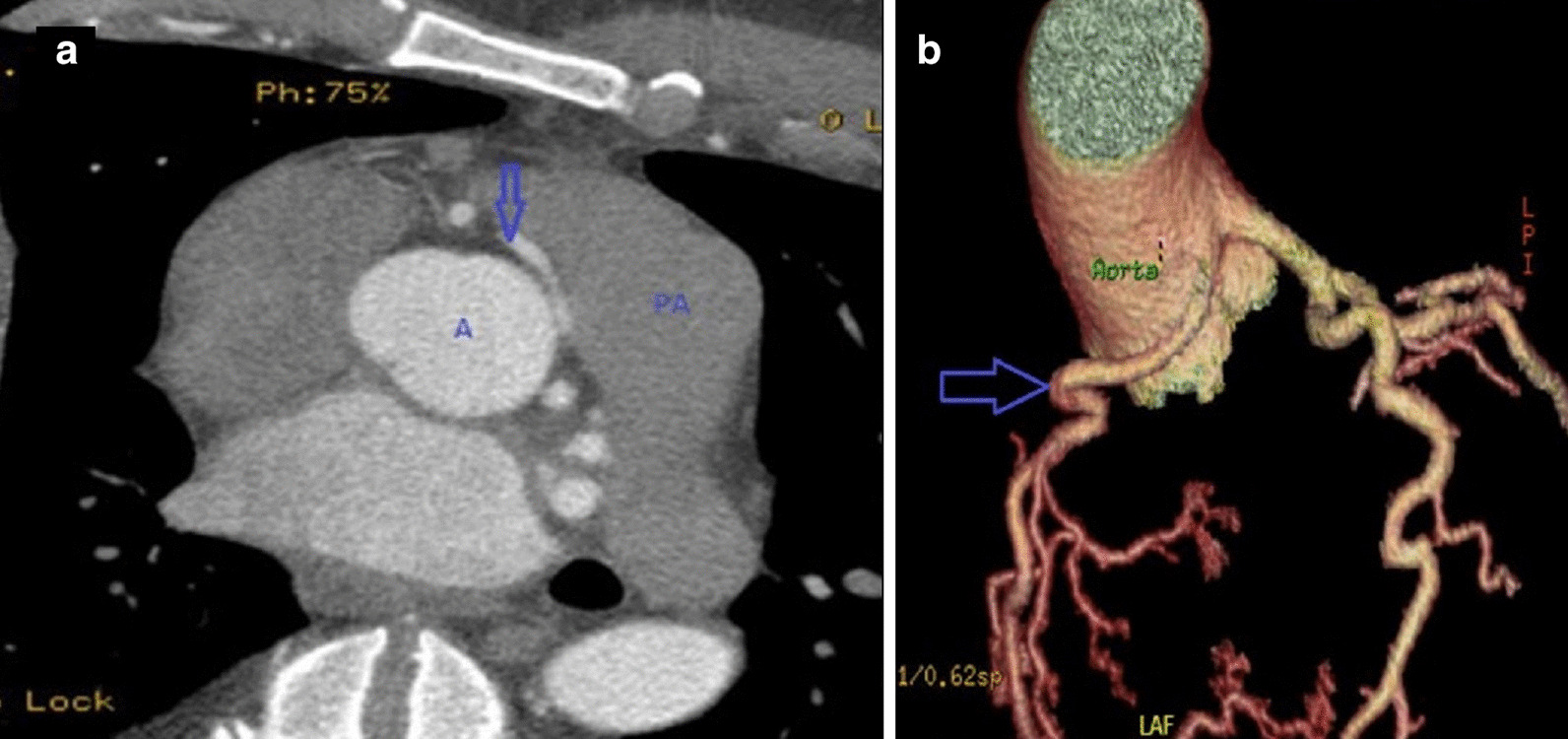
**Case 6.** Coronary CT angiogram (CTA). **a** Axial CTA with anomalous right coronary artery (blue arrow) with left coronary cusp origin just anterior to the origin of the left main coronary artery. **b** Volume rendered CTA with anomalous right coronary artery (blue arrow)

Lab analysis revealed sub-therapeutic prothrombin time and international normalized ratio (INR). Initial work up with coronary angiography revealed trivial coronary artery disease and possible congenital coronary anomaly. TTE on the day after admission demonstrated a LV apical aneurysm with mural thrombus formation. Chest X-ray showed a calcified nodule at the cardiac apex, which correlates to the calcified intramural thrombus. Subsequently, coronary CT angiography (Fig. 23) revealed a potentially malignant, anomalous right coronary artery (RCA) with left coronary cusp origin just anterior to the origin of the left main coronary artery. This anomalous vessel coursed transmurally between the ascending aorta and pulmonary artery; thus, there is a significant risk for vessel impingement both in the transmural course during systole and between the aorta and the pulmonary artery.

TTE with an ultrasound enhancing agent showed mid-LV hypertrophy and an apical LV aneurysm with an apical mass suspicious for a thrombus.

Subsequent CMR showed mid LV hypertrophy consistent with HCM and LV apical aneurysm with intramural thrombus. LGE imaging revealed full thickness scarring in the LV apex. Ultimately, an automated implantable cardioverter defibrillator (ICD) was placed, and the patient was sent home with oral anticoagulation and antiarrhythmic therapy. Patient was counseled and recommended to undergo genetic testing for HCM.

### CMR findings

**Fig. 24:**
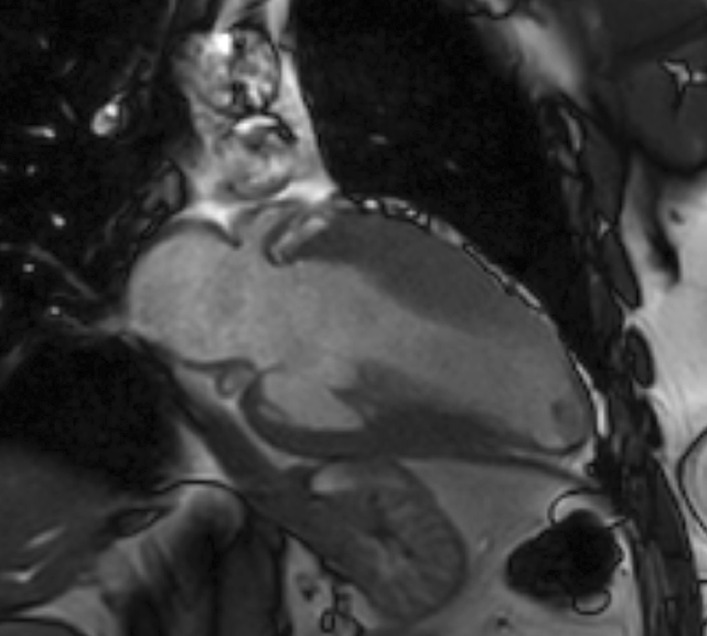
**Case 6.** Cine bSSFP 2 chamber end-diastole. Mid LV thickening with apical aneurysm

**Fig. 25:**
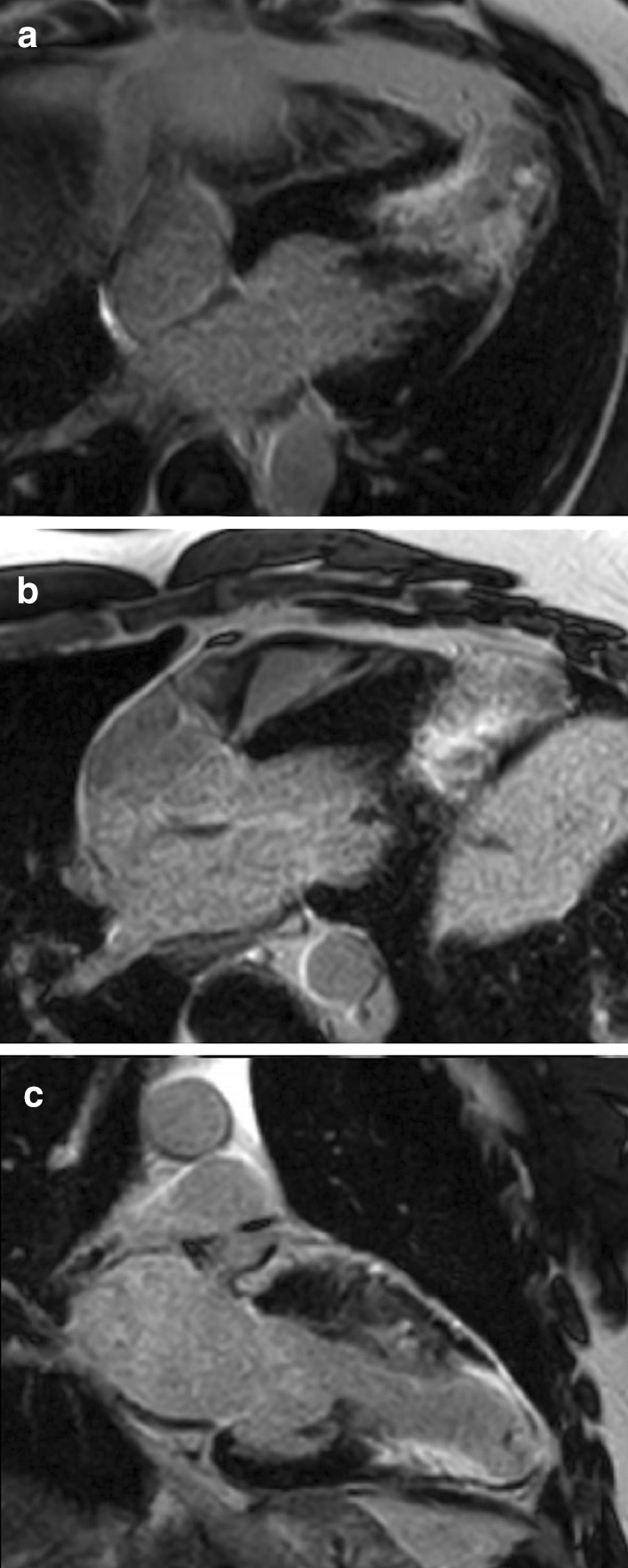
**Case 6.** LGE. 4 chamber view (**a**), 3 chamber view (**b**), and 2 chamber view (**c**) with dense scar of the mid to apical LV

The CMR bSSFP cine images revealed an LVEF of 60% with an apical LV aneurysm measuring 3.9 × 3.2 cm, with moderate hypertrophy of the mid-ventricular myocardium measuring 1.6 cm in maximal thickness consistent with HCM. A 1.2 × 1.0 cm lesion was noted within the apical aneurysm, consistent with intramural thrombus (Additional file 33: Movie S33, Additional file 34: Movie S34, Fig. 24). Also, LGE images revealed near full thickness scarring of the apical LV segments (Fig. 25).

### Conclusion

We report a case of malignant VT with three plausible causes: anomalous RCA with a potentially malignant course, LV apical scarring with aneurysm formation and HCM. However, the apical scar is the most likely cause of the VT based on the VT tracing (Fig. 22). This case emphasizes the role and importance of CMR in evaluation of new-onset VT as it helped determine the most probable source of this life-threatening arrhythmia and saved the patient from undergoing aggressive surgical intervention like: excision and re-implantation of RCA into the appropriate sinus of Valsalva, coronary artery bypass grafting or trans-aortic unroofing of the RCA since such surgical procedures have considerable morbidity and mortality. Hence, it was reasonable to only proceed to ICD implantation in addition to pharmacologic therapy.

### Perspective

VT is a life-threatening arrhythmia, and along with ventricular fibrillation, is the most common cause of outpatient sudden cardiac death. Ventricular arrhythmia mostly occurs in setting of structural heart diseases like post-myocardial infarction (most frequently), coronary artery anomalies, dilated cardiomyopathy, HCM, arrhythmogenic right ventricular dysplasia, and infiltrative diseases like sarcoidosis in addition to non-structural heart diseases like in long QT syndromes. Hence, in patients with new-onset VT, it is vital to pinpoint the source of the aberrant rhythm to prevent the recurrence of potentially life-threatening arrhythmias. As was true in this case, multi-modality imaging plays a major role in evaluation and management of this arrhythmia.

The following will be a discussion of the three potential origins:

1. Anomalous RCA
  While most of coronary artery anomalies are not associated with myocardial ischemia and clinical events, some anomalies can potentially result in myocardial ischemia depending on the location and course of the anomaly; thus, they can manifest with angina pectoris, syncope, myocardial infarction and VT; however, coronary anomalies are considered an under-diagnosed cause of sudden cardiac death (SCD) in young athletes [34].
  Anomalies of RCA include ectopic origin from the right sinus of Valsalva, posterior sinus of Valsalva and left sinus of Valsalva. Our patient has ectopic RCA originating from left sinus of Valsalva which has an incidence rate of 0.03% to 0.92% on coronary angiograms [35]. In our patient, the long intramural course of the anomalous RCA in addition to its inter-arterial course between the aorta and pulmonary artery can potentially result in compression especially during exercise and decreased blood flow to myocardium potentiating ischemic ventricular arrhythmia.
  Coronary anomalies can be seen on echocardiography, angiography, coronary CT and CMR with coronary CT being the clinical standard due to its widespread availability, ease of use, high spatial resolution and very short acquisition time [36].
2. Left ventricular apical scar
  Ventricular scar or fibrosis most commonly forms as a consequence of myocardial infarction, but it can be also caused by non-ischemic insults like myocarditis. These scars are composed of fibrous tissue with intervening cardiomyocytes, and because fibrous tissue is unexcitable, it represents a substrate for re-entry circuits leading to ventricular arrhythmia. As was true in this case, LGE in CMR is the gold-standard imaging for diagnosis of myocardial scar. Normal and viable myocardium appears black on LGE imaging while myocardial scar appears bright or enhanced. Myocardial scar detected on imaging is considered a strong predictor for cardiac death and major adverse cardiac events [37].
  In our patient, the apical scarring was thought to be related to either HCM or less likely thrombotic or embolic event causing infarction in the distal left anterior descending coronary artery (LAD) territory given patient’s history of anti-phospholipid syndrome in setting of sub-therapeutic anticoagulation. It is thus important that the patient be continued on anticoagulation as he would be at risk for further embolic phenomenon.
3. Hypertrophic cardiomyopathy
  HCM is the most common cause of SCD in young athletes. It is mostly asymmetric with most cases having involvement of the basal inter-ventricular septum. However, hypertrophy can exclusively involve the apex, or mid-portion or posterior wall of the LV [38].
  Diagnosis of HCM can be made by demonstrating a wall thickness > 15 mm in one more LV myocardial segments by any cardiac imaging modality, not explained solely by loading conditions [39]. Although the patient in this case has chronic kidney disease and hypertension, neither of these diseases can explain the LV hypertrophy demonstrated on the patient’s imaging as it is focal and asymmetrical (involving mid-segment of the inter-ventricular septum).

Although TTE is considered a good assessment tool for LV wall thickness and LV outflow tract pressure gradient, CMR should be considered for accurate assessment of LV wall thickness in patients with poorly visualized LV regions on echocardiogram, and particularly in HCM cases that are confined to one or two segments of LV like apical or anterolateral variants because of its superior characteristics including lack of attenuation, great spatial resolution in addition to its ability to image in any plane [39]. Also, LGE CMR plays an important prognostic role in predicting adverse cardiovascular events among HCM patients [40].

The CMR of Case 6 (Additional file CMR Link, https://www.cloudcmr.com/9457-1973-5768-0185/).

## Case 7: New murmur in an asymptomatic 10-year-old

### Clinical history

**Fig. 26:**
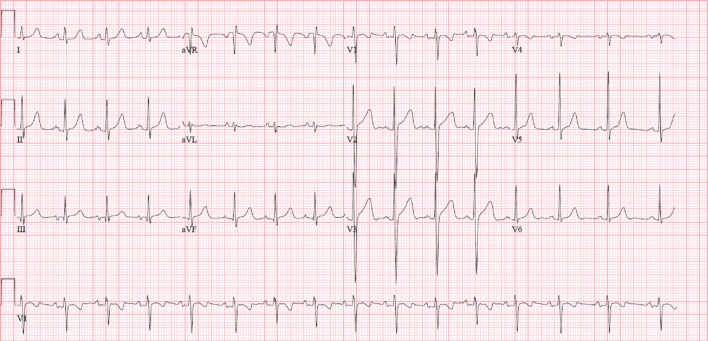
**Case 7.** Twelve lead ECG. LA enlargement present

An asymptomatic 10-year-old male visited his pediatrician for his regular attention deficit and hyperactivity disorder follow-up. A new systolic ejection murmur was heard. Chest X-ray showed an abnormal aortopulmonary window with enlargement of the LA shadow. ECG showed P-mitrale (M-shaped P-waves) in lead II and biphasic P-waves in lead V1, suggesting LA enlargement (Fig. 26). These results prompted a referral to pediatric cardiology. Physical examination revealed a dynamic precordium, a loud first heart sound, a II/VI systolic ejection murmur variable in intensity with inspiration, and an early, high pitched diastolic plop sound at the apex after the second heart sound. TTE (Additional file 35: Movie S35, Additional file 36: Movie S36) revealed a large, mobile, homogeneous mass in a dilated LA. The mass appeared to be pedunculated, but CMR was sought for further investigation.

### CMR findings

**Fig. 27:**
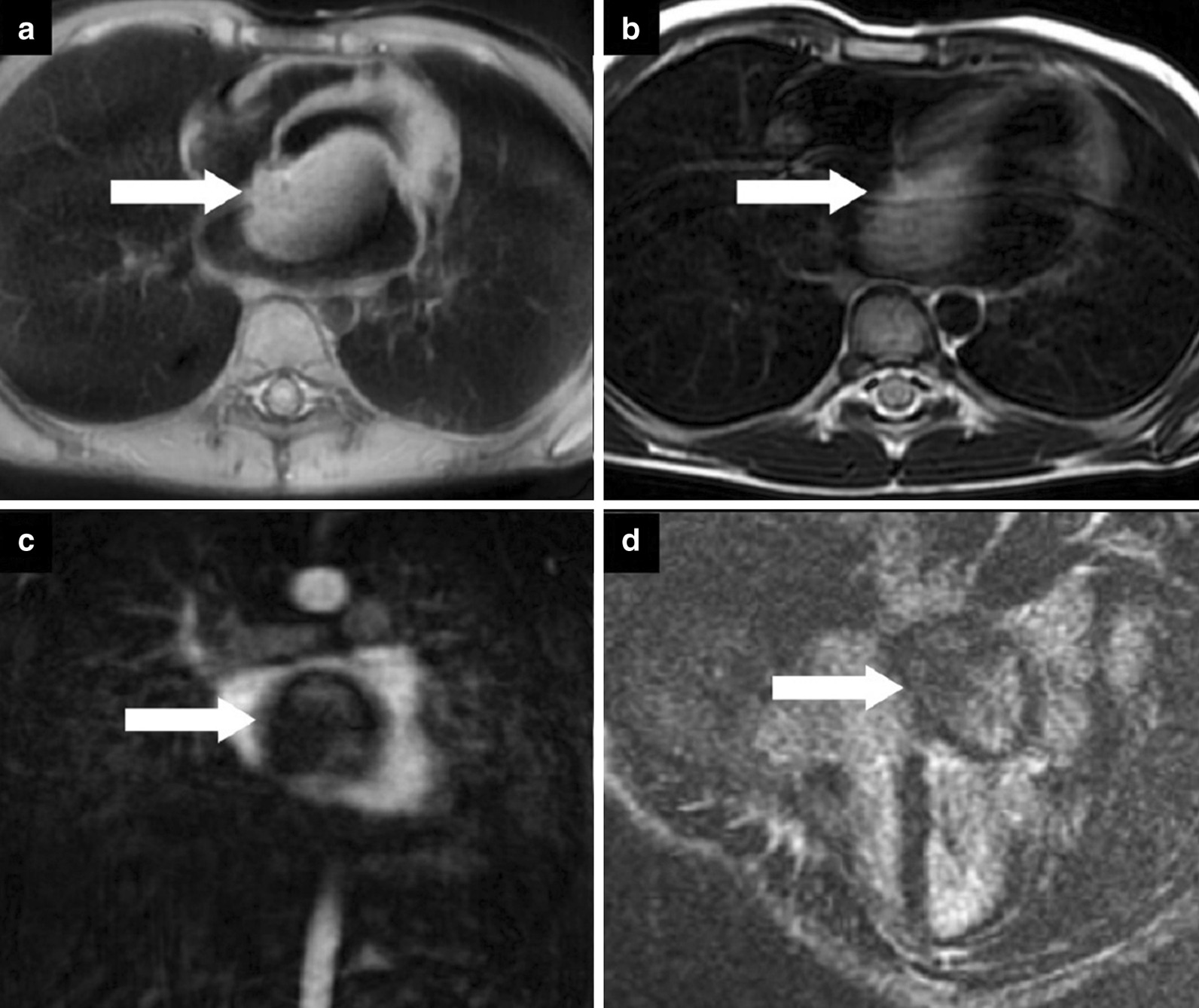
**Case 7.** Variable CMR techniques to evaluate the cardiac mass. **a** Axial T1 double inversion recovery with fat saturation. A large hyperintense mass in LA (white arrow). **b** Axial T2 double inversion recovery image. Hyperintense large LA mass (white arrow). **c** Coronal first pass angiogram. No mass enhancement present (white arrow). **d** Myocardial delayed enhancement four chamber view. Variable intensity of the mass, which could be an India ink artifact (white arrow)

The cine bSSFP short axis view clearly demonstrates a large, smooth, pedunculated and mobile mass (Additional file 37: Movie S37). T1 with fat saturation and T2 imaging both showed hyperintensity (Fig. 27). There was also hypointensity on first pass myocardial perfusion (Fig. 27). All of the CMR findings were in keeping with a probable diagnosis of a benign myxoma [33]. On myocardial LGE (Fig. 27) there was variable intensity, which could be an India ink artifact. With a higher inversion time, a myxoma would appear homogenous. Determining the diagnosis of a LA myxoma, exact location of the stalk, proximity of the myxoma to the pulmonary veins (Additional file 38: Movie S38), and the effects of the tumor on the mitral valve (Additional file 39: Movie S39) were integral in preparation for surgery.

### Conclusion

**Fig. 28:**
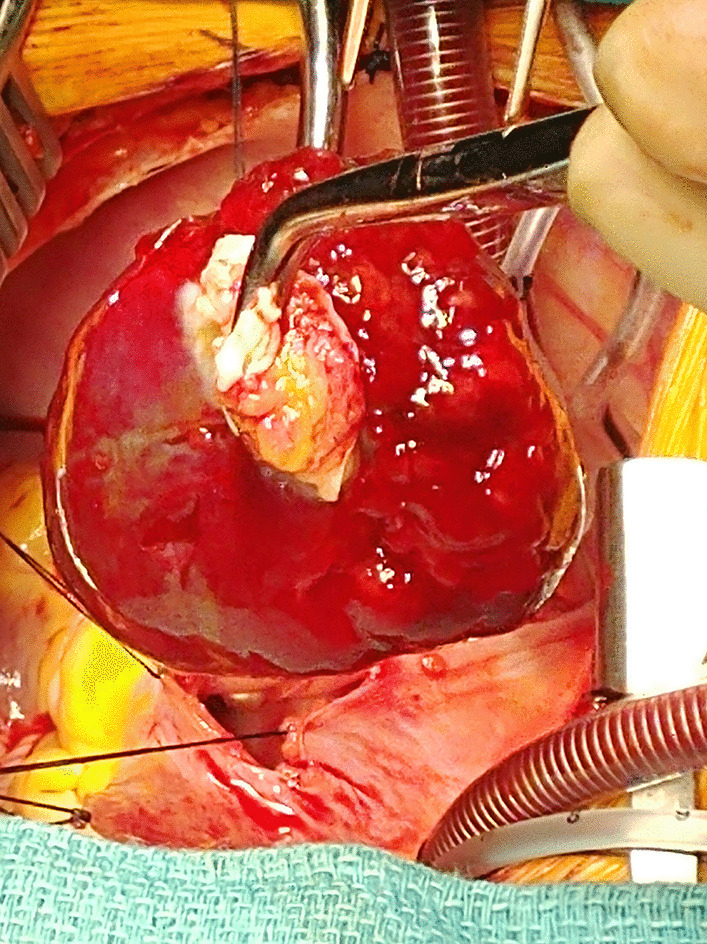
**Case 7.** Operating room picture. Removal of myxoma by the LA stalk

**Fig. 29:**
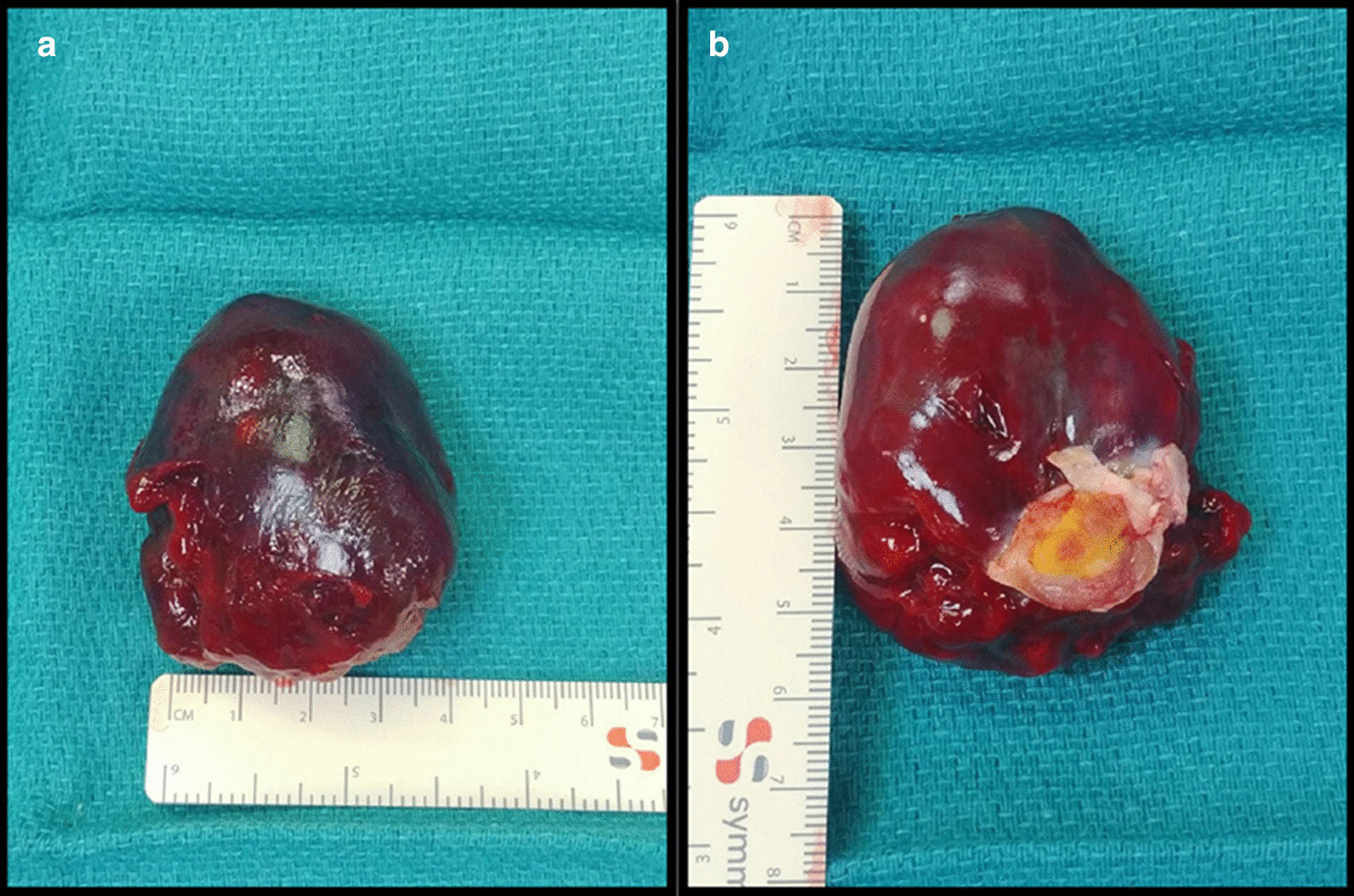
**Case 7.** Resected tumor gross specimen. **a** Myxoma on a surgical towel. **b** Myxoma stalk easily seen. The entire myxoma measures approximately 6 cm × 4 cm

Intra-operatively, after being placed on cardiopulmonary bypass, the LA tumor was resected through the RA by excising the fossa ovalis. The tumor measured approximately 6 cm x 4 cm (Figs. 28, 29) with clear demarcation of the stalk’s attachment to the septum secundum and apex of the fossa ovalis between the pulmonary veins. An atrial septum repair with a pericardial patch was completed. Pathology confirmed the diagnosis of a myxoma. The patient had an uncomplicated post-operative course and was doing well in follow-up at 1 year.

This case demonstrates the importance of CMR in the diagnosis and management of cardiac tumors in children especially in the rare occurrence of a solitary tumor. In our case, the cardiac tumor was easily seen on TTE, but the actual diagnosis could only be suspected. CMR findings characterized the tumor as an atrial myxoma and although CMR has been shown to be highly accurate at differentiating between benign and malignant lesions, pathology remains the diagnostic gold standard [41]. Most importantly, CMR helped to pinpoint the location of the stalk, which was instrumental for complete resection of the tumor without collateral damage to the pulmonary veins or mitral valve.

### Perspective

Cardiac tumors in children have a reported incidence of 0.027–0.08%, most of which are benign [33]. A differential for cardiac tumors may include: myxoma, fibroma, rhabdomyoma, malignancy, thrombus, teratoma, lipoma, and vascular tumors. CMR, along with the clinical correlation, are helpful tools when predicting the diagnosis of solid cardiac tumors. Location and different signal characteristics on commonly used CMR techniques of bSSFP, T1 and T2 fast spin echo, myocardial perfusion, and LGE are highly predictive of particular tumors. For example, a thrombus is often mural or intraluminal with hypointensity on all sequences [33].

Cardiac myxomas are often diagnosed in adults and presentation in children is very rare, generally presenting as part of a genetic syndrome [42–45]. Myxomas develop most commonly in the LA (60%), followed by the RA (28%) and rarely in the ventricles [46]. They typically arise near the fossa ovalis and more rarely from the atrial free wall close to the pulmonary veins or from the atrioventricular valves [46].

The uniqueness of this case stems from the patient’s young age and relatively benign presentation. Large myxomas, like the one in this patient, often present with systemic embolization, mitral valve obstruction or constitutional symptoms [44, 46]. An important consideration when children present with a cardiac myxoma is to screen for possible genetic causes i.e. Carney complex [48]. Carney complex is a rare autosomal dominant syndrome associated with pigmented lesions of the skin and mucosa as well as various types of myxomas and endocrine tumors [47]. The syndrome is due to mutations of the PRKAR1A gene in more than 70% of cases [47]. Other genetic causes are not well understood. Recurrence of the tumor is common in multiple and familial cases [44]. In this case, genetic testing revealed no syndrome as a cause for the myxoma.

The CMR of Case 7 (Additional file CMR Link, https://www.cloudcmr.com/6957-1973-6878-0178/).

## Case 8: Value of CMR in diagnosis and prognostication in a case of relapsing eosinophilic granulomatosis with polyangiitis with myocardial involvement

### Clinical history

A 23-year-old migrant native of Zimbabwe presented with a 10 day history of flu-like symptoms, myalgias, chest pain, palpitations, Troponin I of 1.8 mcg/l (normal < 0.040) and peripheral eosinophilia of 3.42 × 10^9^/l (normal < 0.6 × 10^9^/l). He had been diagnosed with anti-neutrophil cytoplasmic antibody (ANCA) negative EGPA with cardiac involvement 2 years prior, in 2015. Having been managed well on oral methotrexate 25 mg weekly and weaning dose of oral prednisolone, relapse occurred once his prednisolone was decreased to 4 mg daily.

Cardiac investigations were carried out including ECG which demonstrated normal sinus rhythm, normal axis, no PR depression. TTE showed a mildly dilated LV cavity with normal regional/global systolic function. The 3D LVEF was 67%. Trivial loculated pericardial effusions without evidence of hemodynamic compromise (GLS = 21%, RV systolic pressure (RVSP) = 21 mmHg). Endomyocardial biopsy had been carried out in 2015 and was normal, showing no morphological abnormality, no fibrosis or fibrin. No myocyte damage or necrosis was noted. No granulomata or giant cells and no evidence of vasculitis.

Ultimately, his diagnosis of EGPA was made in 2016 following extensive investigation when he presented with dyspnea, chest and abdominal pain on a background of adult onset asthma. He was found to have bilateral pleural effusions and a raised peripheral blood eosinophil count. Diagnosis was ultimately made based on biopsy of skin lesions (which developed in early 2016) and gastrointestinal tract which demonstrated an eosinophilic vasculitis pattern. Cardiac involvement at initial presentation included pericardial effusion as outlined above.

### CMR findings

**Fig. 30:**
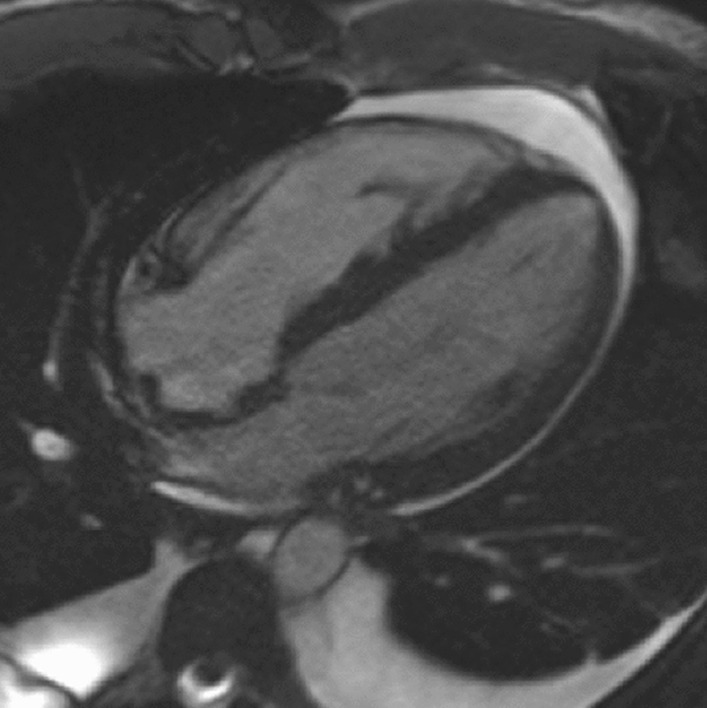
**Case 8.** Cine SSFP 4 chamber. Initial CMR with moderate pericardial effusion

CMR imaging (1.5 T, multiplanar cine bSSFP, T2 double inversion recovery imaging with and without fat saturation and multi-planar LGE) was carried out at first presentation in 2015 showing a moderate pericardial effusion (Fig. 30), without definite myocardial edema or LGE. LVEF was low normal at 53%. This was followed by a normal cardiac biopsy with no evidence of vasculitis/necrosis or fibrosis.

**Fig. 31:**
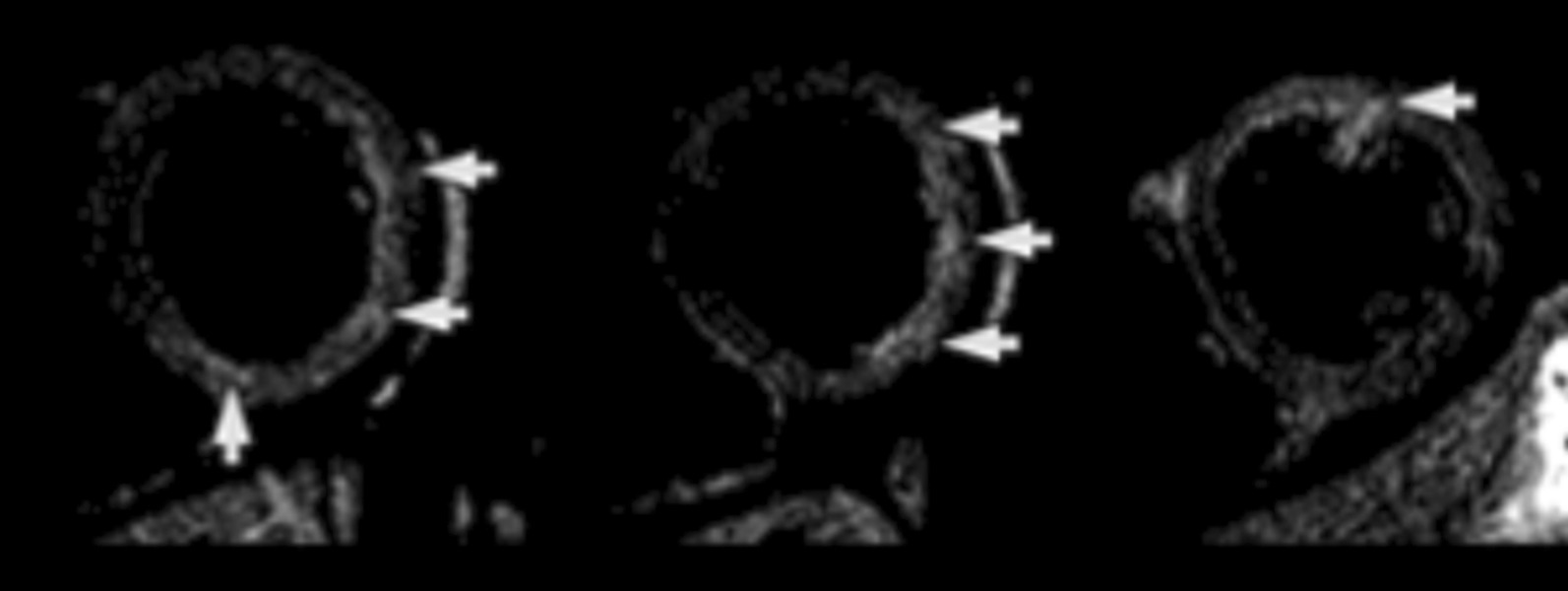
**Case 8.** Short axis T2 double inversion recovery sequences. Myocardial enhancement suggesting edema (white arrows)

**Fig. 32:**
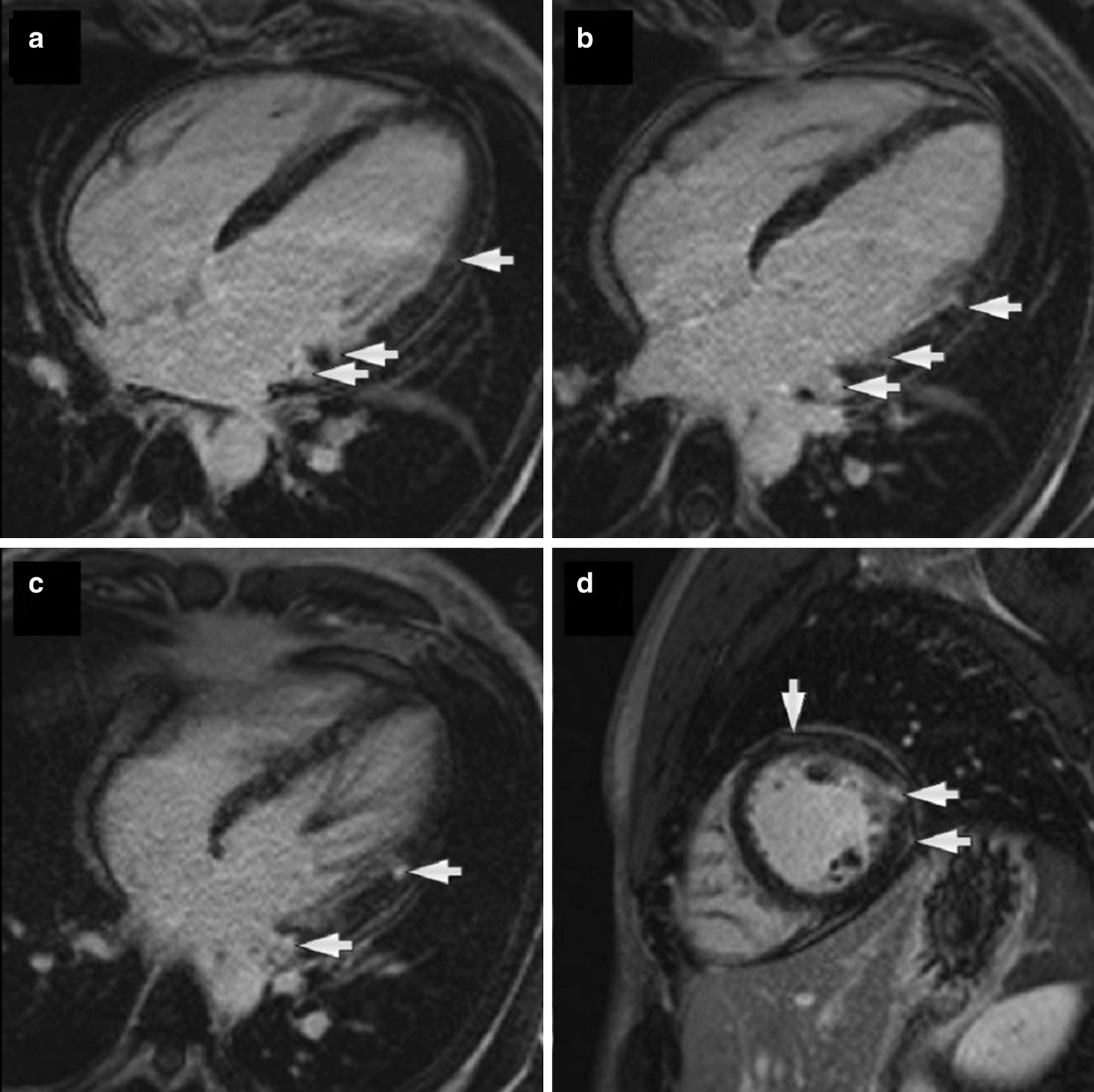
**Case 8.** LGE images. **a**–**c** Four chamber stack with endocardial enhancement throughout the LV myocardium, particularly the lateral LV (white arrows). **d** Short axis mid image with myocardial LGE of the inferolateral wall (white arrows)

**Fig. 33:**
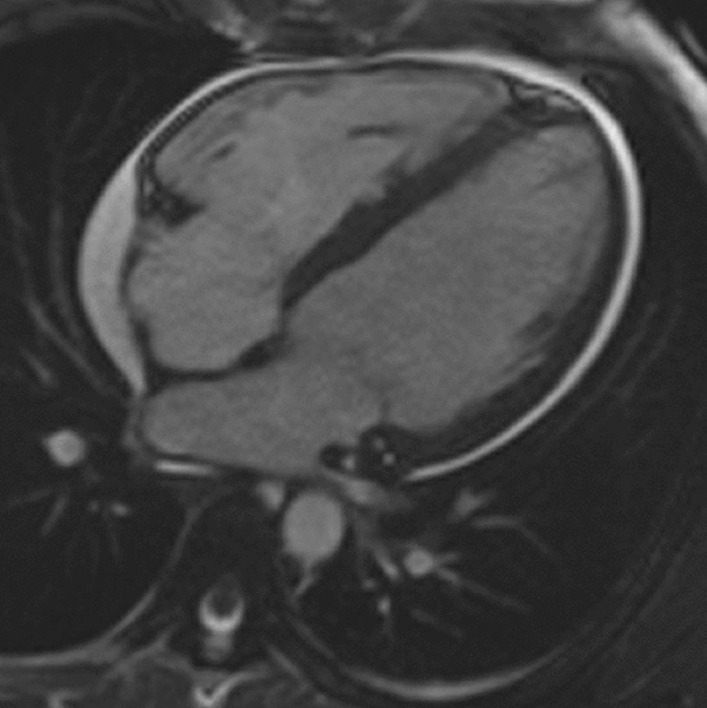
**Case 8.** Four chamber bSSFP cine image. Small pericardial effusion without significant pericardial thickening or pericardial enhancement. There was no evidence of ventricular interdependence. Interval resolution of the bilateral small pleural effusions had occurred

CMR was repeated in April 2018 upon re-presentation which now showed multifocal myocardial hyper-intensity in the basal to mid anterior, lateral and inferior LV walls on T2 double inversion recovery (DIR) sequences suggesting edema (Fig. 31: A three image composite of sequential basal and a mid LV short axis images with a narrow window) and multifocal midwall and sub-epicardial LGE scattered throughout the LV myocardium, particularly the lateral LV wall (Fig. 32). Relative sparing of the apical segments was noted. There was a small pericardial effusion without significant pericardial thickening or pericardial enhancement with no evidence of ventricular interdependence. Interval resolution of the bilateral small pleural effusions had occurred. (Fig. 33) Overall, the findings were consistent with myocarditis.

### Conclusion

This case highlights the importance of CMR in the diagnosis of myocardial involvement in a biopsy negative case of EGPA.

Myocardial involvement was proven on CMR when TTE findings were unhelpful. It also avoided the requirement for repeat cardiac biopsy thereby obviating the necessity for an invasive procedure. Accurate diagnosis allowed for appropriate prognostication and ultimately guided therapy. The patient was subsequently treated with intravenous methylprednisolone and rituximab therapy with excellent clinical response over 6 months and continues to be followed up under the care of rheumatology.

### Perspective

EGPA (previously known as Churg-Strauss vasculitis; see also Case #1) is a rare, vasculitic condition which can affect small and medium sized arteries. It is associated with adult onset asthma, allergic rhinitis and a peripheral blood eosinophilia. It can be associated with multi-organ involvement including cardiac as demonstrated above. Approximately 40–60% of patients with EGPA have positive ANCA [49]. ANCA negative patients, as in this case, tend to exhibit more eosinophil tissue infiltration with higher incidence of myocardial involvement and pulmonary infiltrates. Coronary arteritis and myocarditis are the main causes of morbidity and mortality [50].

Findings of myocarditis on CMR can include increases in T2 signal intensity and increased native myocardial T1 mapping relaxation times consistent with myocardial edema, increase in early myocardial contrast enhancement relative to skeletal muscle consistent with hyperemia, and presence of LGE consistent with necrosis or scarring [51]. CMR allows for tissue characterization in myocarditis specifically diagnosing local edema and demonstrating LGE. Specificity of myocarditis diagnosis by CMR is as high as 91% in some studies and this non-invasive technique may be used in clinical context to avoid invasive cardiac biopsy [52]. Lake Louise Criteria may assist in the diagnosis of myocarditis. At least one T2 based criterion, global or regional increase of myocardial T2 relaxation time or an increased signal intensity in T2w CMR images with one T1 based criterion, increased myocardial T1 time, ECV, or LGE is required for diagnosis. Of note however, discrete lesions may be more difficult to detect after the first few days as the T2 hyperintensity signal becomes gradually more homogenous as edema and inflammation becomes more diffuse within the myocardium [52].

Cardiac involvement in EGPA is associated with poor prognosis. Prognostication is based on the number of organs involved and scoring systems such as the five factor score rely heavily on this for prognostication and to guide treatment [53].

CMR was pivotal in this particular case as diagnosis of myocardial involvement demonstrated a changing clinical picture and allowed for prognostication in the setting of relapsing EGPA. This ultimately highlighted failure of maintenance therapy and guided treatment response.

## Supplementary information

**Additional file 1: Movie S1.** TTE apical 4 chamber view. Systolic obliteration of the LV apex. Although LV apical diastolic thickness was only minimally increased, apical hypertrophic cardiomyopathy (HCM) remained a concern because of suspected LV apical foreshortening.

**Additional file 2: Movie S2.** Cine bSSFP LV 2 chamber.

**Additional file 3: Movie S3.** Cine bSSFP LV 3 chamber.

**Additional file 4: Movie S4.** Cine bSSFP LV 4 chamber.

**Additional file 5: Movie S5.** Rest perfusion imaging of LV basal-, mid- and apical- short axis. Subtle subendocardial hypo-perfusion predominately in mid to apical wall segments.

**Additional file 6: Movie S6.** Rest perfusion imaging 4-chamber view. Subtle subendocardial hypo-perfusion predominately in mid to apical inferoseptal and anterolateral wall segments.

**Additional file 7: Movie S7.** Post-treatment resting perfusion imaging of LV base, mid and apical short axis. Complete resolution of previously noted subtle subendocardial hypo-perfusion.

**Additional file 8: Movie S8.** Post-treatment resting perfusion imaging 4-chamber view. Complete resolution of previously noted subtle subendocardial hypo-perfusion.

**Additional file 9: Movie S9.** Displacement Encoding with Stimulated Echoes (DENSE) sequence mid short axis.

**Additional file 10: Movie S10.** Four chamber cine bSSFP. Normal LV function and absence of mass in the LV cavity.

**Additional file 11: Movie S11.** Basal short axis cine bSSFP. Normal LV function and absence of mass in the LV cavity.

**Additional file 12: Movie S12.** Mid short axis cine bSSFP. Normal LV function and absence of mass in the LV cavity.

**Additional file 13: Movie S13.** Apical short axis cine bSSFP. Normal LV function and absence of mass in the LV cavity.

**Additional file 14: Movie S14.** Cine bSSFP axial aortic arch. Low signal intensity mass in the aortic arch.

**Additional file 15: Movie S15.** Cine bSSFP sagittal aortic arch. Low signal intensity mass in the aortic arch, entering and occluding the brachiocephalic trunk.

**Additional file 16: Movie S16.** T1 fast spin echo sagittal aortic arch. The thrombus had a short T1 consistent with methemoglobin.

**Additional file 17: Movie S17.** T2 fast spin echo sagittal aortic arch. The thrombus had a short T1 consistent with methemoglobin.

**Additional file 18: Movie S18.** Coronal CMR angiogram.

**Additional file 19: Movie S19.** Long inversion time (600 ms) delayed enhancement sagittal aortic arch.

**Additional file 20: Movie S20.** Axial black blood stack demonstrating widespread masses involving the chest and abdomen.

**Additional file 21: Movie S21.** Axial cine SSFP inferior. Complex mediastinal mass infiltrating the left pulmonary veins, extending to the LA and compromising mitral valve inflow.

**Additional file 22: Movie S22.** Axial cine SSFP mid. Complex mediastinal mass infiltrating the left pulmonary veins, extending to the LA. Remaining cardiac chambers are free from infiltration.

**Additional file 23: Movie S23.** Axial cine SSFP superior. Complex mediastinal mass causing near complete occlusion of the left pulmonary artery.

**Additional file 24: Movie S24.** Axial first pass perfusion. Hypovascular nature of the tumor with faint enhancement.

**Additional file 25: Movie S25.** Axial first pass perfusion. Infiltration of the left pulmonary artery.

**Additional file 26: Movie S26.** Fetal echocardiogram four chamber view. Single, large, homogeneous, hyperechoic bi-atrial mass.

**Additional file 27: Movie S27.** Fetal echocardiogram four chamber view with color Doppler. No evidence of obstruction from the large bi-atrial mass.

**Additional file 28: Movie S28.** TTE four chamber view. Large homogeneous, hyperechoic mass mainly in the right atrium.

**Additional file 29: Movie S29.** TTE four chamber with color Doppler. No LA obstruction from the atrial mass.

**Additional file 30: Movie S30.** TTE subcostal sagittal color compare. Large homogeneous, hyperechoic mass arising from the Eustachian valve. Patent foramen ovale with left to right shunting.

**Additional file 31: Movie S31.** Axial cine SSFP stack. A right atrial mass attached to the Eustachian valve with no evidence of obstruction.

**Additional file 32: Movie S32.** Axial first pass perfusion. Contrast uptake of the atrial mass present.

**Additional file 33: Movie S33.** Cine bSSFP 4 chamber. Mid LV thickening with apical aneurysm.

**Additional file 34: Movie S34.** Cine bSSFP 3 chamber. Apical LV aneurysm.

**Additional file 35: Movie S35.** TTE parasternal long axis with color Doppler. Obstruction of mitral valve inflow from the mass with mitral valve insufficiency.

**Additional file 36: Movie S36.** TTE four chamber. The LA mass extending past the mitral valve annulus during diastole.

**Additional file 37: Movie S37.** Short axis cine bSSFP. A large, relatively smooth, pedunculated mass attached to the interatrial septum.

**Additional file 38: Movie S38.** Axial cine bSSFP. A large hypointense LA mass likely attached to interatrial septum at the superior edge of the fossa ovalis close to the pulmonary vein entrance.

**Additional file 39: Movie S39.** Cine bSSFP four chamber view showing large variably hypointense mass bulging into the LV during diastole causing dynamic mitral stenosis.

## Data Availability

All data and materials are available.

## Abbreviations

ANCA: Anti-neutrophil cytoplasmic antibody
ASE: American Society of Echocardiography
bSSFP: Balanced steady state free precession
CMR: Cardiovascular magnetic resonance
CRP: C-reactive protein
CT: Computed tomography
DENSE: Displacement encoding with stimulated echoes
DIR: Dual inversion recovery
DMF: Diffuse myocardial fibrosis
ECG: Electrocardiogram
ECV: Extracellular volume fraction
EGPA: Eosinophilic granulomatosis with polyangiitis
EMS: Emergency medical services
ESR: Erythrocyte sedimentation rate
GDMT: Guideline directed medical therapy
GLS: Global longitudinal strain
HARP: Harmonic phase
HCM: Hypertrophic cardiomyopathy
HF: HEART failure
ICD: Implantable cardioverter defibrillator
INR: International normalized ratio
IVC: Inferior vena cava
LA: Left atrium/left atrial
LAD: Left anterior descending coronary artery
LBBB: Left bundle branch block
LGE: Late gadolinium enhancement
LV: Left ventricle/left ventricular
LVEF: Left ventricular ejection fraction
MOLLI: Modified Look-Locker inversion recovery
MRI: Magnetic resonance imaging
PSIR: Phase sensitive inversion recovery
RA: Right atrium/right atrial
RCA: Right coronary artery
RV: Right ventricle/right ventricular
RVEF: Right ventricular ejection fraction
RVSP: Right ventricular systolic pressure
SCD: Sudden cardiac death
SCMR: Society for Cardiovascular Magnetic Resonance
SMA: Superior mesenteric artery
STIR: Short tau inversion recovery
T1w: T1 weighted
T2w: T2 weighted
TEE: Transesophageal echocardiogram
TTE: Transthoracic echocardiogram
VT: Ventricular tachycardia
