# Supplementary figures and images for: Society for Cardiovascular Magnetic Resonance 2019 Case of the Week series
Source: J Cardiovasc Magn Reson. 2021 Apr 1;23:44. doi: 10.1186/s12968-020-00671-7 (PMC8015162; doi:10.1186/s12968-020-00671-7)

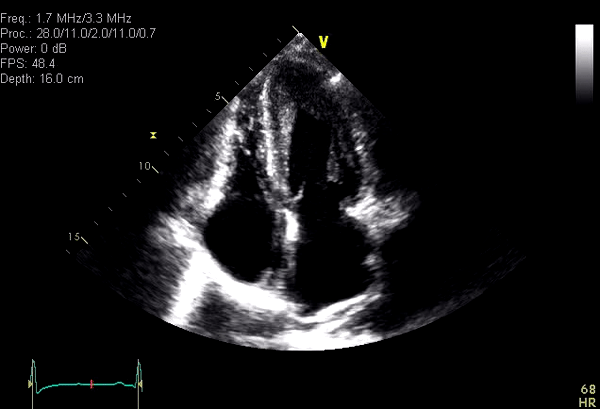

Supplement: Supplementary file 1 — Additional file 1: Movie S1. TTE apical 4 chamber view. Systolic obliteration of the LV apex. Although LV apical diastolic thickness was only minimally increased, apical hypertrophic cardiomyopathy (HCM) remained a concern because of suspected LV apical foreshortening. [file 12968_2020_671_MOESM1_ESM.gif]

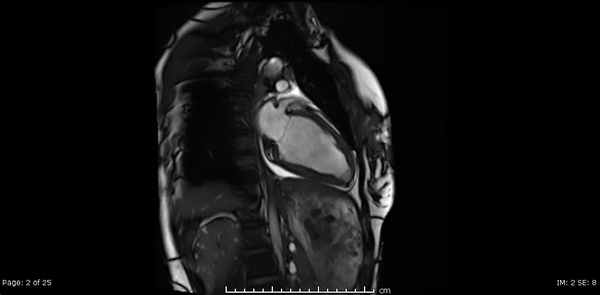

Supplement: Supplementary file 2 — Additional file 2: Movie S2. Cine bSSFP LV 2 chamber. [file 12968_2020_671_MOESM2_ESM.gif]

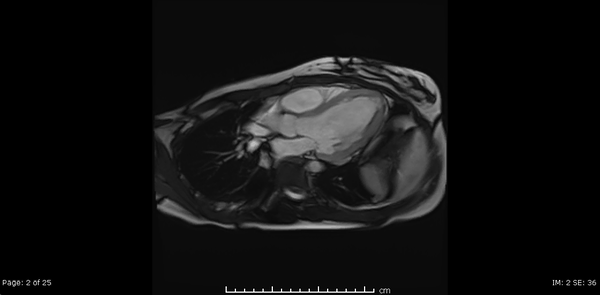

Supplement: Supplementary file 3 — Additional file 3: Movie S3. Cine bSSFP LV 3 chamber. [file 12968_2020_671_MOESM3_ESM.gif]

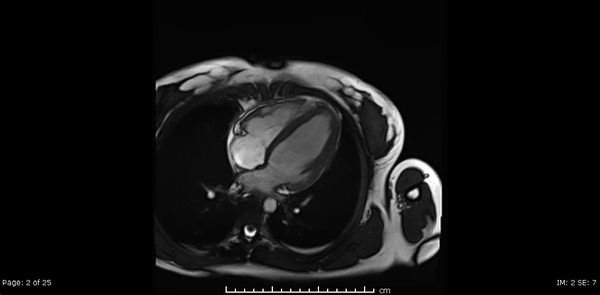

Supplement: Supplementary file 4 — Additional file 4: Movie S4. Cine bSSFP LV 4 chamber. [file 12968_2020_671_MOESM4_ESM.gif]

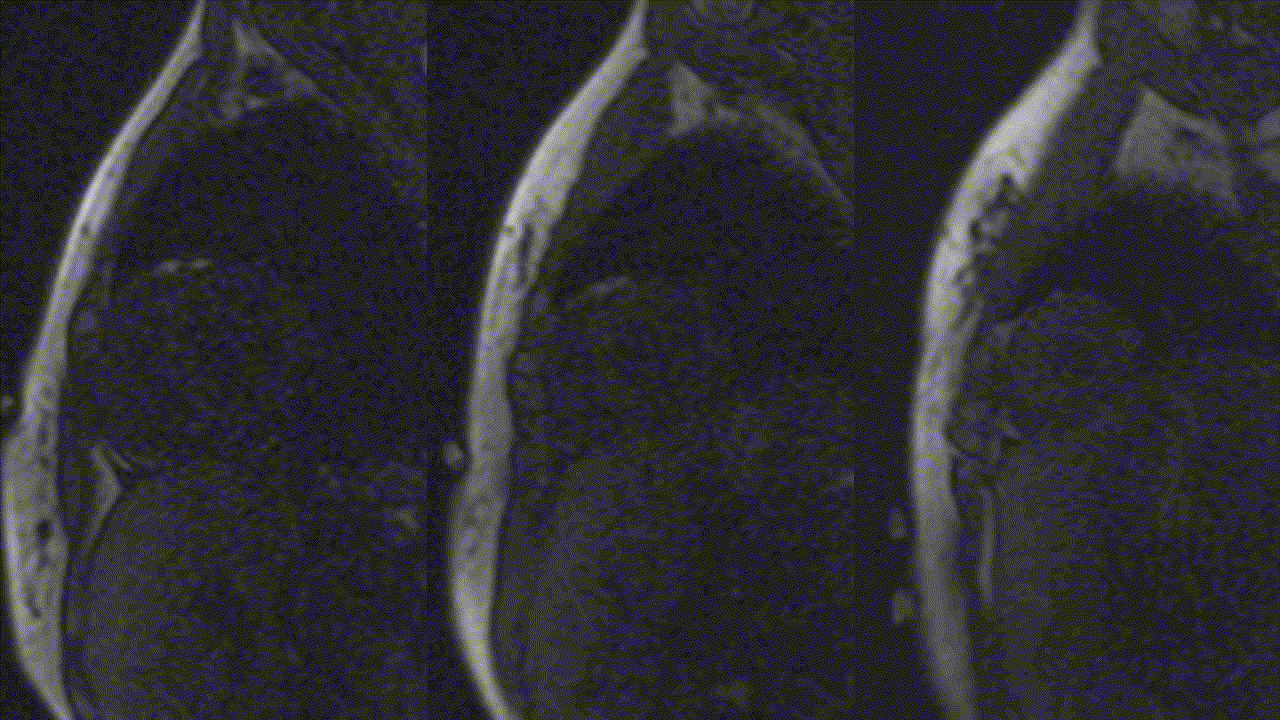

Supplement: Supplementary file 5 — Additional file 5: Movie S5. Rest perfusion imaging of LV basal-, mid- and apical- short axis. Subtle subendocardial hypo-perfusion predominately in mid to apical wall segments. [file 12968_2020_671_MOESM5_ESM.gif]

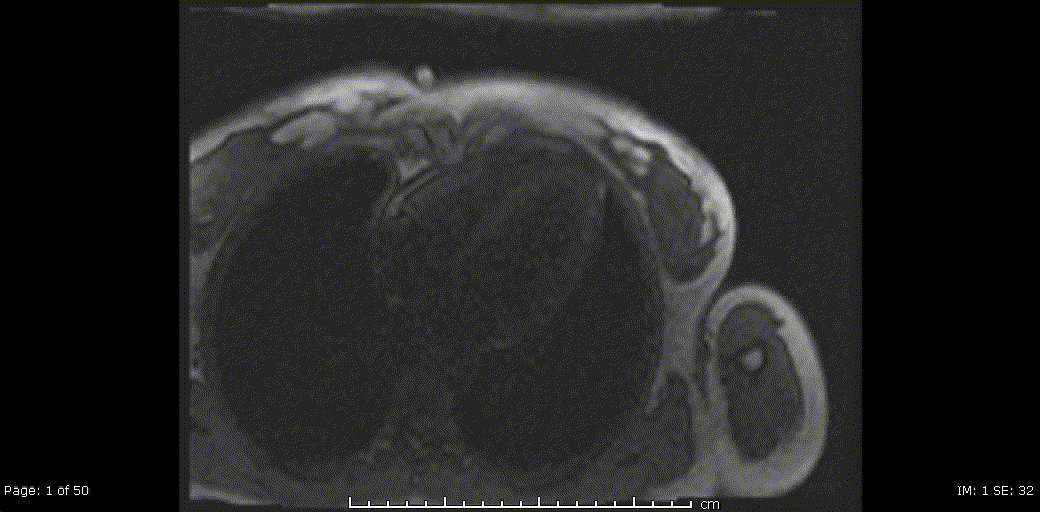

Supplement: Supplementary file 6 — Additional file 6: Movie S6. Rest perfusion imaging 4-chamber view. Subtle subendocardial hypo-perfusion predominately in mid to apical inferoseptal and anterolateral wall segments. [file 12968_2020_671_MOESM6_ESM.gif]

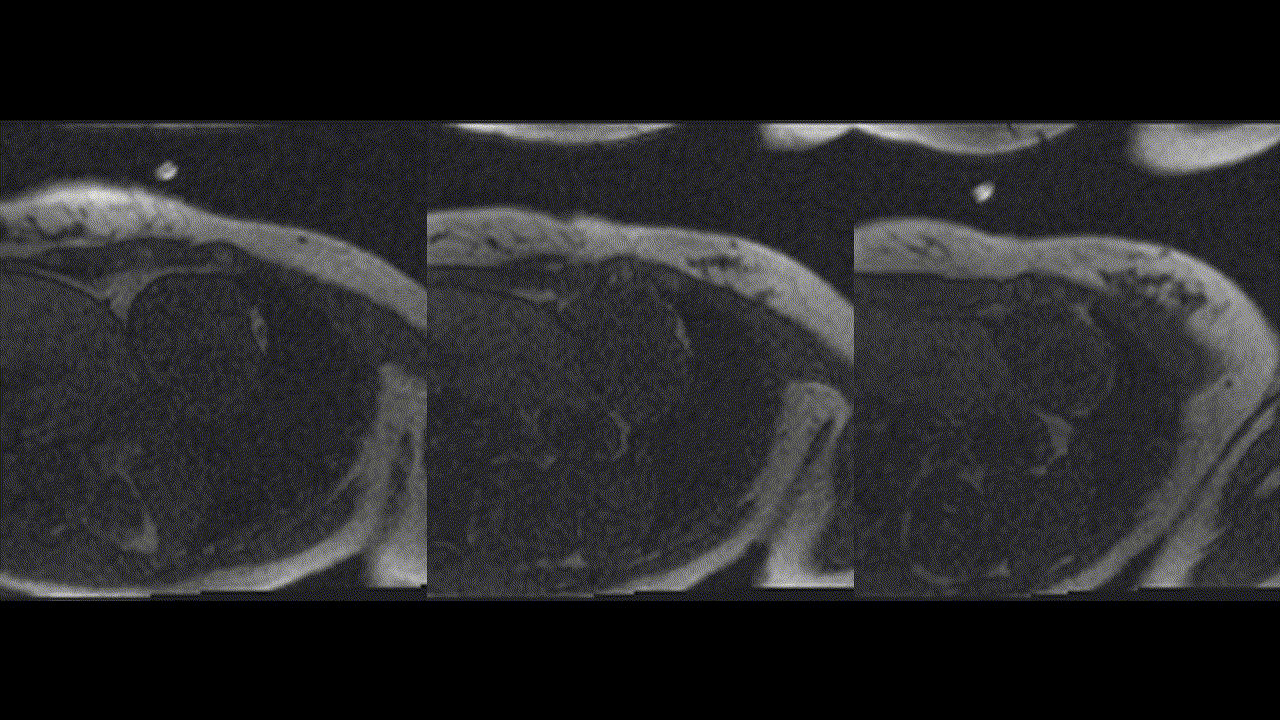

Supplement: Supplementary file 7 — Additional file 7: Movie S7. Post-treatment resting perfusion imaging of LV base, mid and apical short axis. Complete resolution of previously noted subtle subendocardial hypo-perfusion. [file 12968_2020_671_MOESM7_ESM.gif]

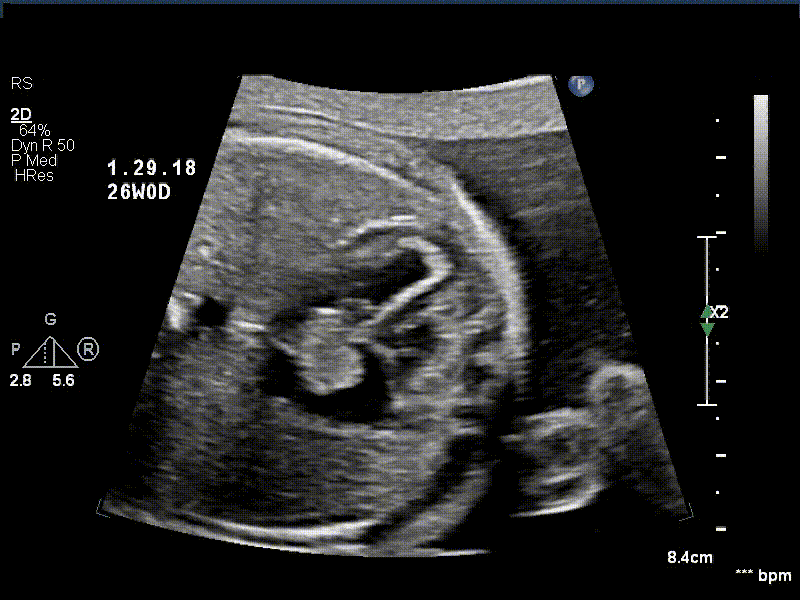

Supplement: Supplementary file 26 — Additional file 26: Movie S26. Fetal echocardiogram four chamber view. Single, large, homogeneous, hyperechoic bi-atrial mass. [file 12968_2020_671_MOESM26_ESM.gif]

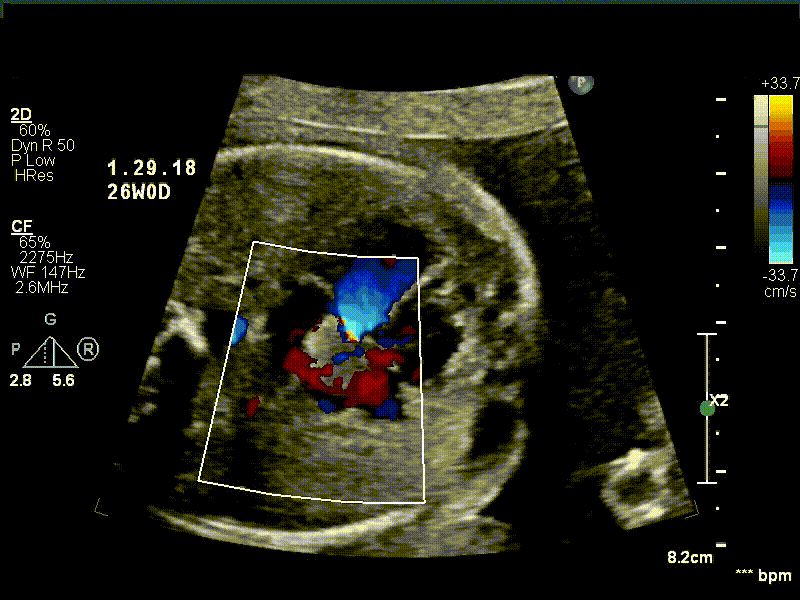

Supplement: Supplementary file 27 — Additional file 27: Movie S27. Fetal echocardiogram four chamber view with color Doppler. No evidence of obstruction from the large bi-atrial mass. [file 12968_2020_671_MOESM27_ESM.gif]

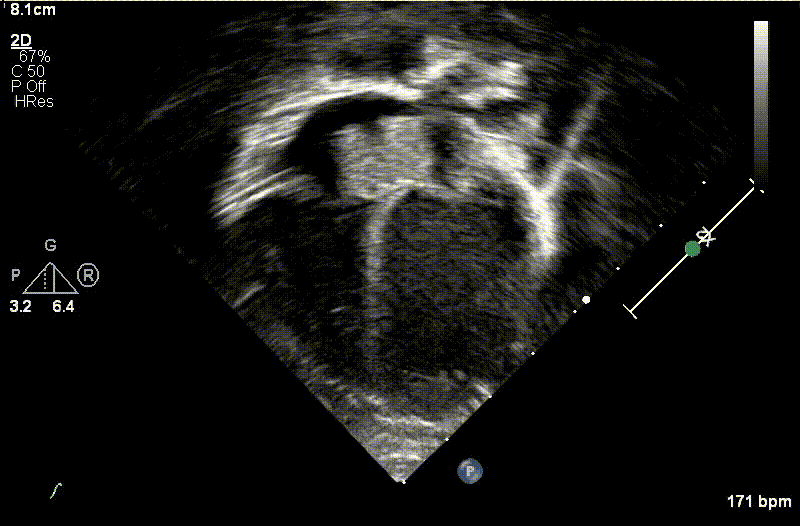

Supplement: Supplementary file 28 — Additional file 28: Movie S28. TTE four chamber view. Large homogeneous, hyperechoic mass mainly in the right atrium. [file 12968_2020_671_MOESM28_ESM.gif]

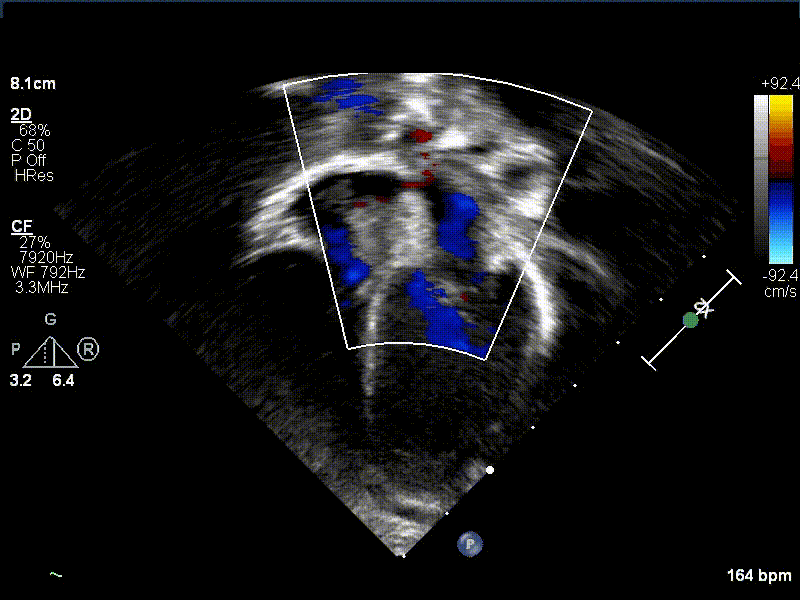

Supplement: Supplementary file 29 — Additional file 29: Movie S29. TTE four chamber with color Doppler. No LA obstruction from the atrial mass. [file 12968_2020_671_MOESM29_ESM.gif]

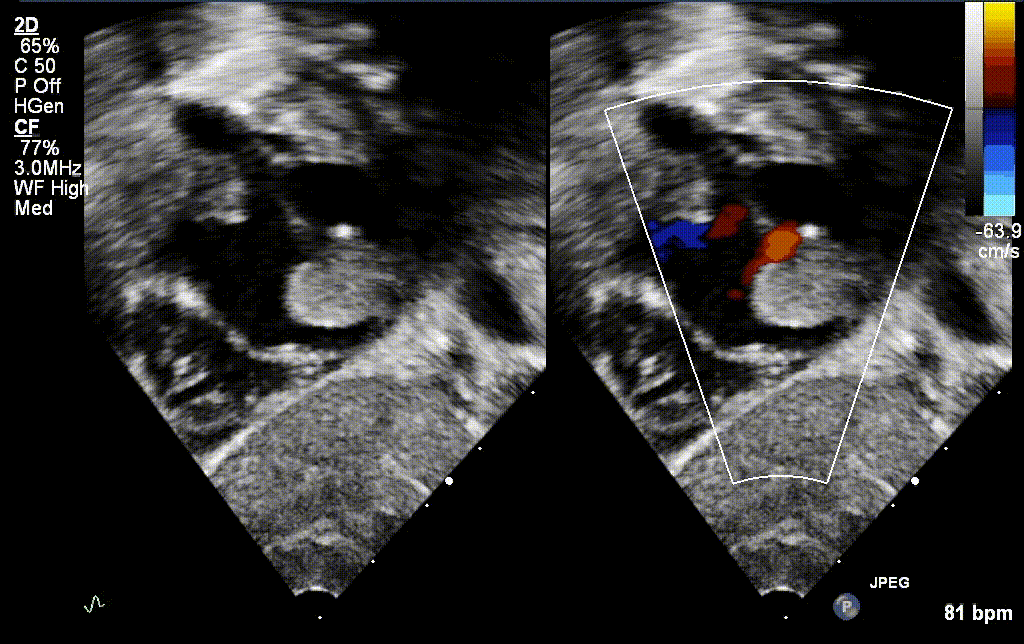

Supplement: Supplementary file 30 — Additional file 30: Movie S30. TTE subcostal sagittal color compare. Large homogeneous, hyperechoic mass arising from the Eustachian valve. Patent foramen ovale with left to right shunting. [file 12968_2020_671_MOESM30_ESM.gif]

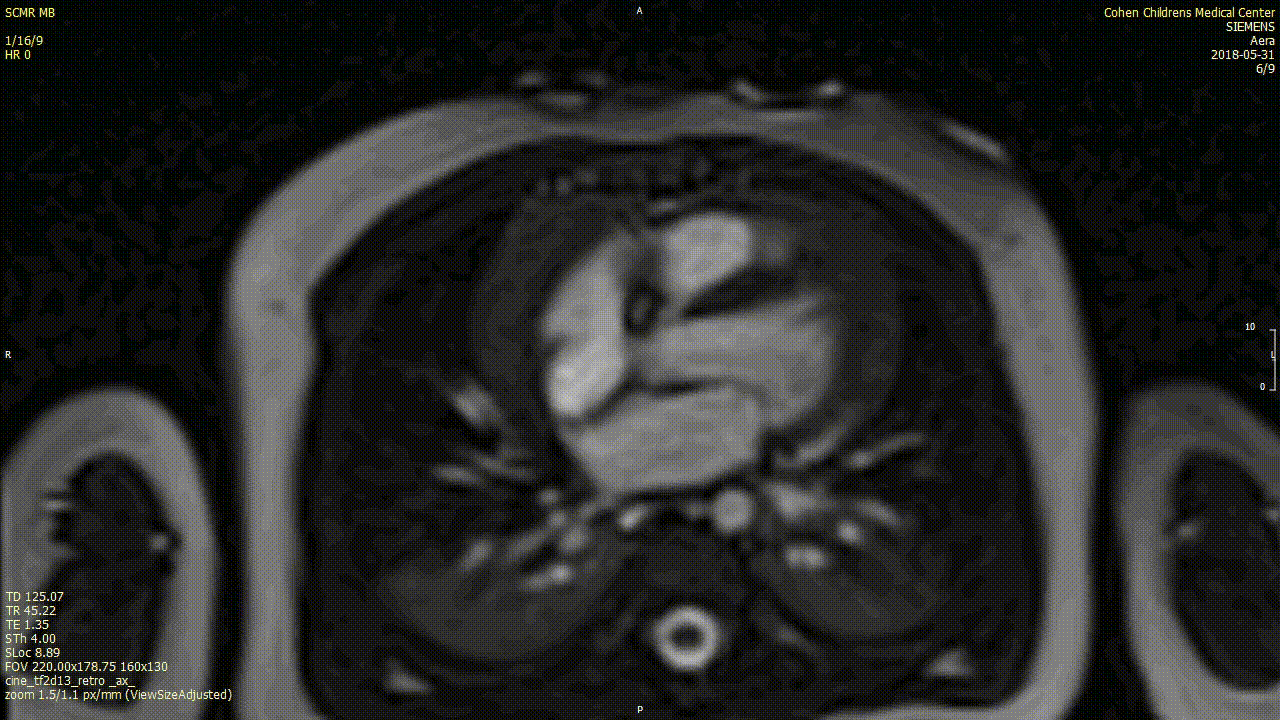

Supplement: Supplementary file 31 — Additional file 31: Movie S31. Axial cine SSFP stack. A right atrial mass attached to the Eustachian valve with no evidence of obstruction. [file 12968_2020_671_MOESM31_ESM.gif]
